# Ultrafast Luminescence Detection with Selective Adsorption of Carbon Disulfide in a Gold(I) Metal−Organic Framework

**DOI:** 10.1002/anie.202413830

**Published:** 2024-12-17

**Authors:** Haruka Yoshino, Masaki Saigo, Takumi Ehara, Kiyoshi Miyata, Ken Onda, Jenny Pirillo, Yuh Hijikata, Shinya Takaishi, Wataru Kosaka, Ken‐ichi Otake, Susumu Kitagawa, Hitoshi Miyasaka

**Affiliations:** ^1^ Institute for Materials Research Tohoku University 2-1-1 Katahira Aoba-ku Sendai 980-8577 Japan; ^2^ Department of Chemistry Faculty of Science Kyushu University Motooka 744 Nishi-ku Fukuoka 819-0395 Japan; ^3^ Department of Chemistry and Biotechnology School of Engineering Department of Materials Chemistry Graduate School of Engineering Nagoya University Furo-cho Chikusa-ku Nagoya 464-8603 Japan; ^4^ Research Center for Net Zero Carbon Society Institute of Innovation for Future Society Nagoya University Furo-cho Chikusa-ku Nagoya 464-8603 Japan; ^5^ Department of Chemistry Graduate School of Science Tohoku University Sendai Miyagi 980-8578 Japan; ^6^ Institute for Integrated Cell-Material Sciences Institute for Advanced Study Kyoto University Kyoto Japan

**Keywords:** Coordination polymers/metal-organic frameworks, aurophilic interactions, luminescence sensing, selective CS_2_ adsorption, host-guest interactions

## Abstract

Although a widely used and important industrial chemical, carbon disulfide (CS_2_) poses a number of hazards due to its volatility and toxicity. As such, the development of multifunctional materials for the selective capture and easy recognition of CS_2_ is one of the crucial issues. Herein, we demonstrate completely selective CS_2_ adsorption among trials involving H_2_O, alcohols, volatile organic compounds (including thiol derivatives), N_2_, H_2_, O_2_, CH_4_, CO, NO, and CO_2_. We also showcase its fine detection using remarkable luminescent response in a gold(I)‐based metal−organic framework (MOF) of {Zn^II^(pz)[Au^I^(CN)_2_]_2_} (pz=pyrazine; **1**) with a two‐fold interpenetration network. *Ex situ* single crystal X‐ray diffraction for **1** and CS_2_‐accommodated **1** suggested that the Au ⋅⋅⋅ Au atoms are not only luminescent centers but also act as interaction sites for CS_2_ modulating the Au ⋅⋅⋅ Au contacts. These experiments revealed the specificity of CS_2_ and how changes in the CS_2_‐induced structure. Based on the obtained structural transformation, **1** exhibited a sensitive detecting ability for CS_2_ with an ultrafast response time of less than 10 s. Moreover, *ex situ* time‐resolved photoluminescence analyses developed in this work implied that CS_2_ varied the energetic relaxation at the excited states related to the luminescent efficiency of the resultant MOF system.

## Graphical abstract

A novel phosphorescent metal‐organic framework (MOF) exhibited a sensitive detecting ability for toxic carbon disulfide (CS_2_) with an ultrafast response time of 10 s at room temperature. *Ex situ* time‐resolved photoluminescence systems developed in this work demonstrated that the manipulation of energetic relaxation at the excited state with CS_2_ adsorption led to the luminescence response with outstanding visibility.

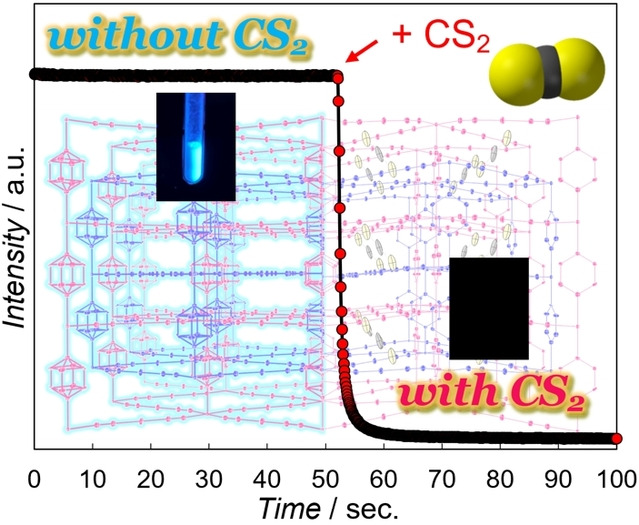

## Introduction

Among the industrial chemicals produced on a large scale, carbon disulfide (CS_2_) is a typical commodity owing to its wide range of use in the production of agricultural pesticides, vulcanizing accelerants, C_1_ synthons, non‐polar solvents, and even as a processing reagent in the manufacture of cellophane and viscose rayon.[^1^, ^2^, ^3^, ^4^] Conversely, the large‐scale production and consumption of CS_2_ lead to numerous issues including environmental risks such as acid rain, organic aerosols, flammability associated with volatility, and radiation reactions involved in the formation of carbon dioxide (CO_2_),[^5^, ^6^, ^7^, ^8^] as well as human risks such as atherosclerosis, neurological and cardiovascular diseases, and peripheral neuropathy.[^9^, ^10^, ^11^, ^12^] Hence, the development of novel functional materials that exhibit high sensitivity and simple detection at room temperature (RT) for CS_2_ with colorless and toxicity is important from the viewpoints of the environmental and human health care.

Luminescent compounds with the ability to detect specific chemicals are attractive research targets owing to their potential applications in highly functional sensors and devices.[^13^, ^14^, ^15^] Recently, coordination polymers (CPs) and metal‐organic frameworks (MOFs) consisting of metal nodes/clusters and organic ligands have attracted considerable interest as platforms for coupling porosity‐related functions and physical properties because of their inherent modularity and flexible structures.[^16^, ^17^, ^18^, ^19^, ^20^, ^21^, ^22^] For instance, robust and chemically stable MOFs, namely [M_2_(dobpdc)] (M=Mg^2+^,Ni^2+^;dobpdc^4−^=4,4’‐dioxidobiphenyl‐3,3’‐dicarboxylate), exhibited SO_2_−driven turn‐on response of luminescence intensity related to the electronic effect associated with the SO_2_ adsorption on to the unsaturated metal sites in the framework.[^23^, ^24^] Various mechanisms have been exploited to develop guest‐responsive luminescent materials so far, such as charge transfer and metal‐centered emission.[^25^, ^26^, ^27^] Notably, employing metallophilic interactions facilitated by Pt(II), Cu(I), Ag(I), and Au(I) complexes is one of the suitable approaches for luminescence sensing because the assembly structures and aggregation/orientation modes of M⋅⋅⋅M units significantly affect the absorption/emission energy of the luminescent materials with metallophilic interactions.[^28^, ^29^, ^30^, ^31^] Hence, porous materials containing M⋅⋅⋅M moieties are expected to achieve significant guest‐dependent luminescent variations through structural changes accompanied by guest adsorption. However, despite the demonstrated hazards of toxic chemicals involving CS_2_, the research on luminescent CPs/MOFs capable of detecting selective capture of toxic gas in real‐time at RT through their photophysical properties within the framework, including the details of host‐guest interactions, adsorption sites, and detection speed, have rarely been unexplored to date due to the instability of the host frameworks.^[32]^

In the present study, we demonstrate the completely selective adsorption and prominent detection of CS_2_ by emission change at RT in a new 3‐D MOF of {Zn^II^(pz)[Au^I^(CN)_2_]_2_} (pz=pyrazine; **1**). The excellent CS_2_‐selectivity along with the ultrafast luminescent response of **1** was expounded by detailed investigations of the structural and emission conversions through vapor/gas adsorption, column breakthrough tests with CS_2_/CO_2_ gas, X‐ray diffraction, and photoluminescence spectroscopy under CS_2_.

## Results and Discussion

**Figure 1:**
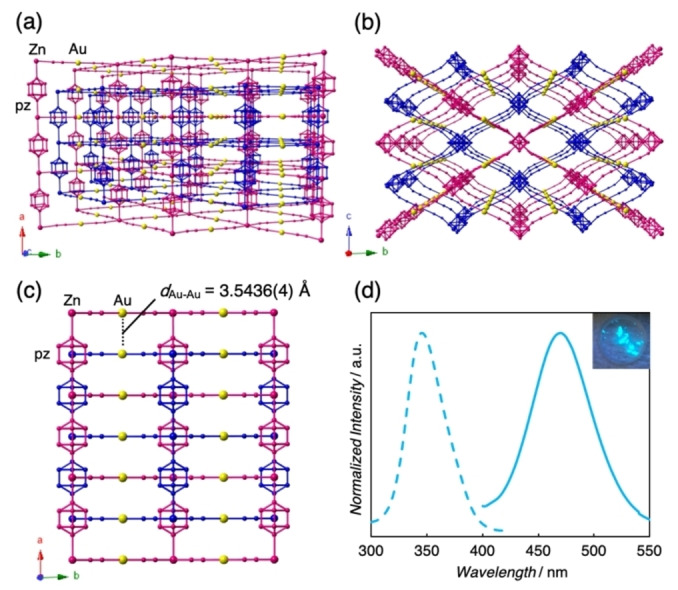
Crystal structure of **1** at 298 K viewed along the (a) *c*‐ and (b) *a*‐axis. Au atoms are represented by yellow spheres and deep blue and pink highlight the two‐fold interpenetration networks. H atoms are omitted for clarity. (c) Dimeric aurophilic interaction distance in **1** at 298 K. (d) Emission (solid line, λ_ex_=365 nm) and excitation (dashed line, λ_em_=471 nm) spectra of **1** at RT. A photograph of **1** under UV irradiation (λ_ex_=365 nm) at RT is shown in the inset.

Single crystals of **1** were synthesized via a slow‐diffusion method using an aqueous solution of ZnCl_2_, pyrazine, and K[Au(CN)_2_]. Experimental details are provided in the Supporting Information (SI). Single crystal X‐ray diffraction (SCXRD) analysis showed that **1** crystallized in the orthorhombic space group *Fmmm*,^[33]^ as summarized in Table S1. The crystal structure of **1** at 298 K showed that the Zn^II^ ions in **1** were equatorially coordinated with the four cyanide nitrogen atoms of the [Au^I^(CN)_2_]_2_ moieties, resulting in the rhombic {Zn^II^[Au^I^(CN)_2_]_2_} grids in the *bc* plane (Figure 1(a) and (b)). Additionally, the bridging pz ligands occupied the remaining axial positions of the Zn^II^ centers as pillars along the (100) direction, giving rise to two‐fold interpenetration 3‐D networks with disordered pz ligands. The Au⋅⋅⋅Au distance in each sub‐lattice within the framework of **1** was 3.5436(4) Å at 298 K (Figure 1(c)). Besides, the obtained SCXRD data and thermogravimetric analysis (TGA) (Figure S4) for **1** indicated the absence of solvent molecules in the framework. The resultant colorless crystals of **1** exhibited light‐blue emission under UV illumination (Figure 1(d)).

Steady‐state optical properties of **1** were investigated, and the corresponding photophysical properties are listed in Table S2. **1** displays a broad emission with a photoluminescence quantum yield (*Φ*_em_) of approximately 7.6 % under excitation at 365 nm (solid line in Figure 1(d)). Moreover, the excitation spectrum of **1** detected at the emission maximum also shows a broad band (dashed line in Figure 1(d)). In addition to the steady‐state spectroscopy, photoluminescence lifetimes (*τ*) at RT were measured and the emission decay curves are presented in Figure S5. The emission decay of **1** was fitted using the triple exponential functions, providing a weighted average *τ* value (*τ*_ave_) of 0.482 μs (Table S2). The sub‐microsecond lifetime of **1** indicates the phosphorescent property with a spin‐forbidden triplet origin, similar to that of previously reported Au(I) complexes containing aurophilic interactions.[^34^, ^35^, ^36^, ^37^] Significantly, density functional theory (DFT) calculations of **1** referring to the SCXRD result at 298 K implied that the highest occupied molecular orbital (HOMO) and the lowest unoccupied molecular orbital (LUMO) were the antibonding characters located between the Au atoms and the π* orbital of the pz ligands, respectively (Figure S8(a)). These results suggested that metal‐metal‐to‐ligand charge transfer (MMLCT) transitions could be caused by a combination of metallophilic interactions and ligands with appropriate π* orbitals.[^30^, ^38^, ^39^]

**Figure 2:**
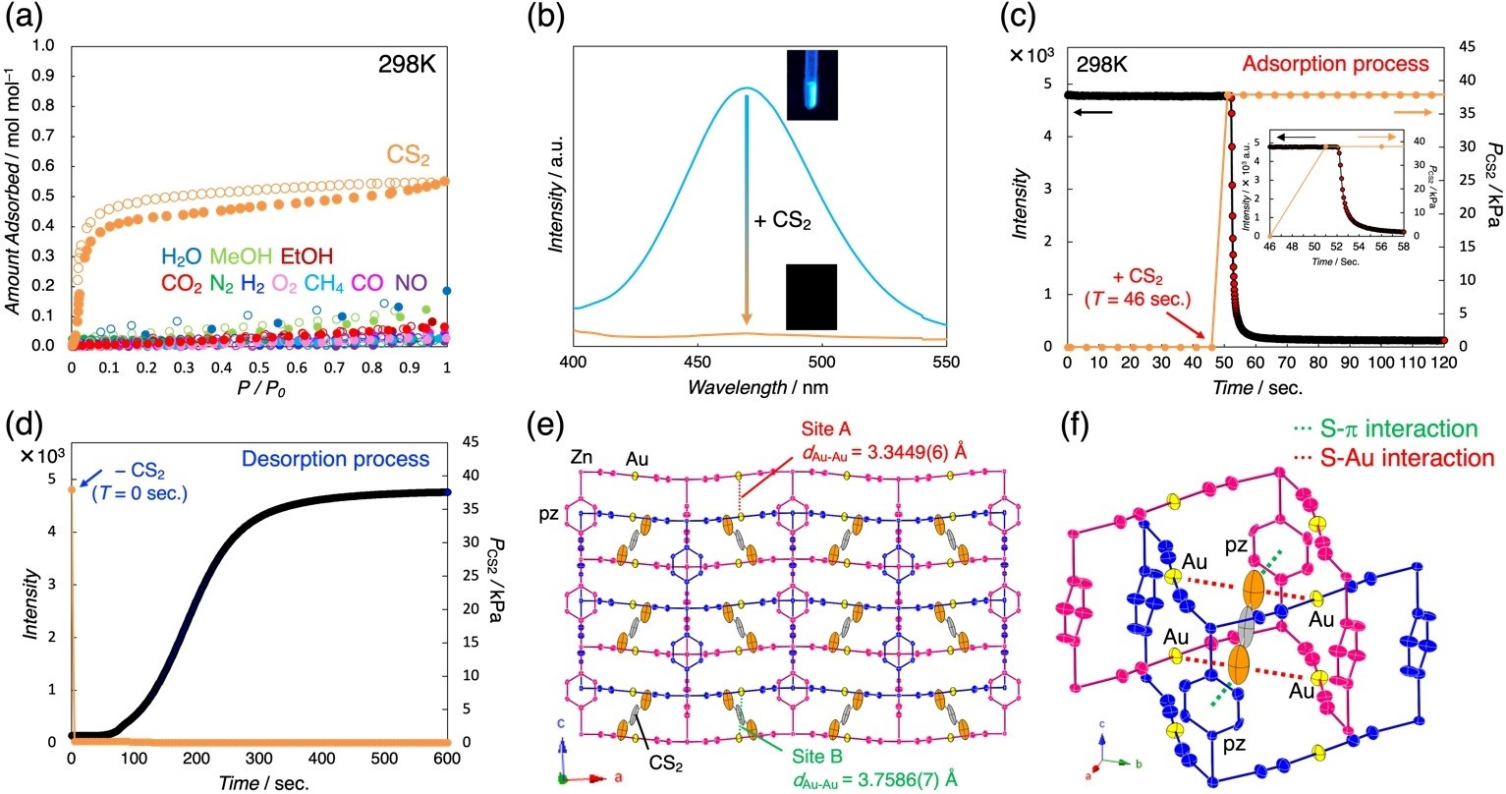
(a) Adsorption and desorption isotherms of **1** for vapor and gas guests at 298 K. The filled and open circles display the adsorption and desorption processes, respectively. (b) *In situ* emission spectra of **1** (light blue) and **1**_**CS_2_** (orange) at 298 K (λ_ex_=365 nm). Time‐course measurements of the luminescence intensity during (c) CS_2_ adsorption and (d) desorption process. Red and blue arrows display the starting points of CS_2_ injection/ejection (valve opening). (e) An ORTEP drawing of the crystal structure for **1**_**CS_2_** at 298 K viewed along the *b*‐axis. Orange and yellow spheres represent S and Au atoms, and deep blue and pink highlight the two‐fold interpenetration networks. H atoms are omitted for clarity. Thermal ellipsoids are shown at the 50 % probability level. (f) Host−guest interactions in the crystal structure of **1**_**CS_2_** at 298 K.

The vapor and gas adsorption measurements were performed to evaluate the guest‐responsivity of **1**. The adsorption/desorption isotherms of **1** for solvent vapors and common gases depicted no adsorption profiles at RT, and the emission color did not change either (Figure 2(a)). Other guest molecules such as acetonitrile, acetone, benzene, and sulfur‐based guests were likewise not impacted on the host structure and emission color due to the weak host‐guest interaction and closed packing structure of **1** (Figure S10). Nevertheless, **1** exhibited an unambiguous selectivity only for CS_2_ with a one‐step adsorption behavior in the low‐pressure region, unlike the other guest molecules including smaller and similarly shaped CO_2_ (Figure 2(a) and Table S3).^[40]^ The adsorbed amount of CS_2_ at 298 K reached 0.5513 mol/mol per unit at *P*/P_0_=0.9929. Hence, the resultant size‐inverse and selective adsorption of **1** for CS_2_ was attributed to effective host‐guest interactions rather than to the simple size effect of the guests (*vide infra*). To further investigate the affinity of CS_2_ for **1**, variable‐temperature (VT) CS_2_ adsorption data were collected.

All VT‐adsorption isotherms showed abrupt adsorption behaviors at a certain relative pressure (*P*/P_0_), following the order: 288 K<298 K<308 K (Figure S9(a)). The adsorption enthalpy (*ΔH*_ads_) of CS_2_ for **1** was obtained by using the Clausius–Clapeyron equation.[^41^, ^42^] The resultant *ΔH*_ads_ value was determined to be approximately 55 kJ/mol in the low‐pressure region (Figure S9(b)). *In situ* observation of the CS_2_‐driven luminescence variation at 298 K was directly monitored by combining a spectrofluorometer and cryostat system with a sample room designed to introduce CS_2_ vapor. Distinct luminescence quenching was observed upon the injection of CS_2_ vapor at *P*_CS2_=37.9 kPa (Figure 2(b)). Surprisingly, time‐course measurements of *in situ* photoluminescence measurement during the CS_2_ adsorption process revealed that the luminescence intensity of the framework rapidly decreased with an increase in time, reaching a plateau with a fast response time of 10 s (Figure 2(c)). The luminescence intensity was returned after CS_2_ desorption under vacuum at 298 K; in contrast to the adsorption process, complete regeneration of guest‐free **1** required approximately 10 min (Figure 2(d)), indicating the host‐guest interactions. Note that heating and vacuum treatment at 373 °C can easily return to **1** in just 1 min (Figure S11). Significantly, **1** also showed a rapid emission response even at low vapor pressure of CS_2_ at 140 Pa, corresponding to the first adsorption point of the adsorption isotherm at 298 K (Figure S12).

*In situ* powder X‐ray diffraction (PXRD) patterns were recorded to understand the CS_2_‐induced structural change in **1** (Figure S6). The CS_2_‐adsorbed state (**1**_**CS_2_**) was obtained by dosing CS_2_ vapor to **1** at RT (see SI for details). **1** underwent a structural transformation to **1**_**CS_2_** involving slight changes in the diffraction positions (Figure S6). Besides, the TGA curve of **1**_**CS_2_** confirmed that the CS_2_ molecules in the pores were removed below 400 K and remained stable until the framework decomposition around 500 K (Figure S4). To comprehend the crystal structure of **1**_**CS_2_** in more detail, *ex situ* SCXRD analysis under CS_2_ vapor was performed. Consequently, the space group of **1**_**CS_2_** was determined to be *Pmma*,^[33]^ where undulating {Zn^II^[Au^I^(CN)_2_]_2_} layers were formed along with a subtle increase in the cell volume upon CS_2_ adsorption (Figure 2e, Figure S1c, d, and Table S1). In addition, the resultant single‐crystal‐to‐single‐crystal transformation from **1** to **1**_**CS_2_** caused a disorder‐order transition of pillar pz ligands associated with the structural stabilization (Figure S13): the carbon atoms of pz ligand in **1**_**CS_2_** were found to be fixed (Figure 2e), while those of **1** were disordered over two general positions with 0.5:0.5 occupancies (Figure 1a). Furthermore, the structural information of **1**_**CS_2_** offered an interpretation of the origin for the CS_2_ adsorbed amount (approximately 0.5 mol/mol per unit) observed in the ads/desorption measurements (Figure 2a). The crystal structure of **1**_**CS_2_** viewed along the *c*‐axis revealed that the CS_2_ molecules were accommodated within half of the sites, whose pz ligands were arranged parallel to the *b*‐axis (Figure S1e). Meanwhile, the other sites with a vertical arrangement on the *b*‐axis inhibited CS_2_ capture due to steric hindrance. Hence, the fix of the position for pz ligands enhances the adsorption capacity of the framework: although the guest‐free **1** formed a nonporous structure, the solvent‐accessible volume of **1**_**CS_2_** was estimated at 3.96 % by using *PLATON/VOID* software.^[43]^ The most notable structural feature of **1**_**CS_2_** was the two types of the Au⋅⋅⋅Au distances (Figure 2e): one is 3.3449(6) Å (site A) and the other is 3.7586(7) Å (site B), which could be vital for the CS_2_‐driven luminescent response. In theory, metallophilic interactions cannot occur if the M⋅⋅⋅M distance is larger than twice the van der Waals radius of the M ions (*i. e*., 3.60 Å for Au⋅⋅⋅Au interactions).[^31^, ^44^] Considering the previous studies, site B (*d*_Au⋅⋅⋅Au_=3.7586(7) Å) does not contain aurophilic interactions. Indeed, the DFT calculation of the site B structure for **1**_**CS_2_** indicated that the HOMO of this state was not σ*_Au−Au_ character (Figure S8c). Thus, the anisotropic structural change of **1** along with CS_2_ adsorption would be one of the key factors for the resultant visual changes through emission quenching.

The host–guest interactions in the crystal structure of **1** _**CS_2_** were further evaluated from the SCXRD results to obtain insights into selective CS_2_ adsorption. Remarkably, two kinds of interactions including S⋅⋅⋅π and S⋅⋅⋅Au contacts were indicated between the S atoms of CS_2_ and pz rings and Au atoms of each sub‐lattice within the framework of **1**, where the corresponding distances were 3.326 Å (S⋅⋅⋅π) and 3.310 Å (S⋅⋅⋅Au), respectively (Figure 2f).[^45^, ^46^] As evidenced by the CS_2_ adsorption profiles and the calculated *ΔH*_ads_ value, these interactions stabilized of the crystal form of **1** _**CS_2_**. The effective interactions between pz ligands and CS_2_ with positive quadrupole moments plausibly resulted in the selective CS_2_ adsorption accompanied by the disorder‐order structural transformation.[^47^, ^48^] Notably, it is worth mentioning that the luminescent center of **1**, the Au atoms, can also act as an interaction site for CS_2_.

**Figure 3:**
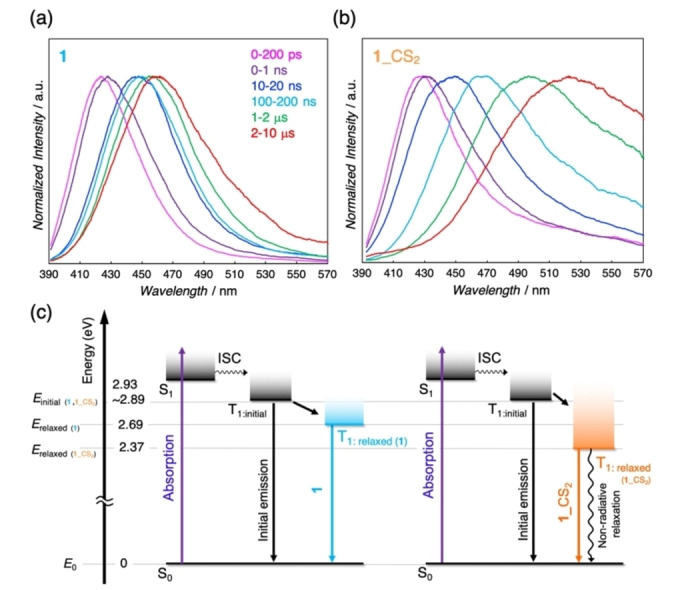
Normalized *ex situ* TR‐PL spectra (λ_ex_=365 nm) of (a) **1** and (b) **1**_**CS_2_** at RT, where pink, purple, deep blue, light blue, green, and red spectra represent 0−200 ps, 0−1 ns, 10−20 ns, 100−200 ns, 1−2 μs, and 2−10 μs time region, respectively. (c) Proposed schematic energy diagram of **1** and **1**_**CS_2_** from the analysis of the TR‐PL spectra.

We studied the impact of the structural differences between **1** and **1** _**CS_2_** at the ground state on their luminescent nature via *ex situ* TR‐PL measurements. Investigating the effects of excited state dynamics on the spectral features is crucial for comprehending the intrinsic properties of luminescent materials.[^49^, ^50^] Therefore, *ex situ* TR‐PL systems under vacuum or CS_2_ vapor conditions were developed in this work to explore the dynamics of **1** and **1** _**CS_2_** at the excited states (details are given in SI). The TR‐PL spectra of **1** and **1** _**CS_2_** from the initial state immediately after excitation (*i. e*. ~200 ps) to the relaxed state (*i. e*. ~10 μs) are shown in Figures 3a and b. In both, the maximum emission energy of the initial state (*E*_init._) was higher than that of the relaxed state (*E*_relax._) because of an energetic stabilization involving vibrational and dielectric relaxation at the excited states. Hence, the energy gap between *E*_init._ and *E*_relax._ (*ΔE*) implies the degree of the electronic relaxation process at the excited states. The obtained *E*_init._, *E*_relax._, and *ΔE* values of **1** and **1** _**CS_2_** are listed in Table S5. The *E*_init._ values of both cases were close, attributing to the fact that the HOMO and LUMO potentially relevant to the observed spectra possess similar characteristics (Figures S8a and b). Nevertheless, the *ΔE* value of **1** _**CS_2_** (0.52 eV) was unambiguously larger than that of **1** (0.24 eV). These results indicated that the nonradiative decay process was enhanced according to the energy gap law (Figure 3c), which was also collaborated by the photophysical properties of **1** and **1** _**CS_2_** (Table S2). Thus, the TR‐PL results demonstrate that CS_2_ adsorption induces significant energetic relaxation at the excited state, which would also contribute to the outstanding luminescence response.

**Figure 4:**
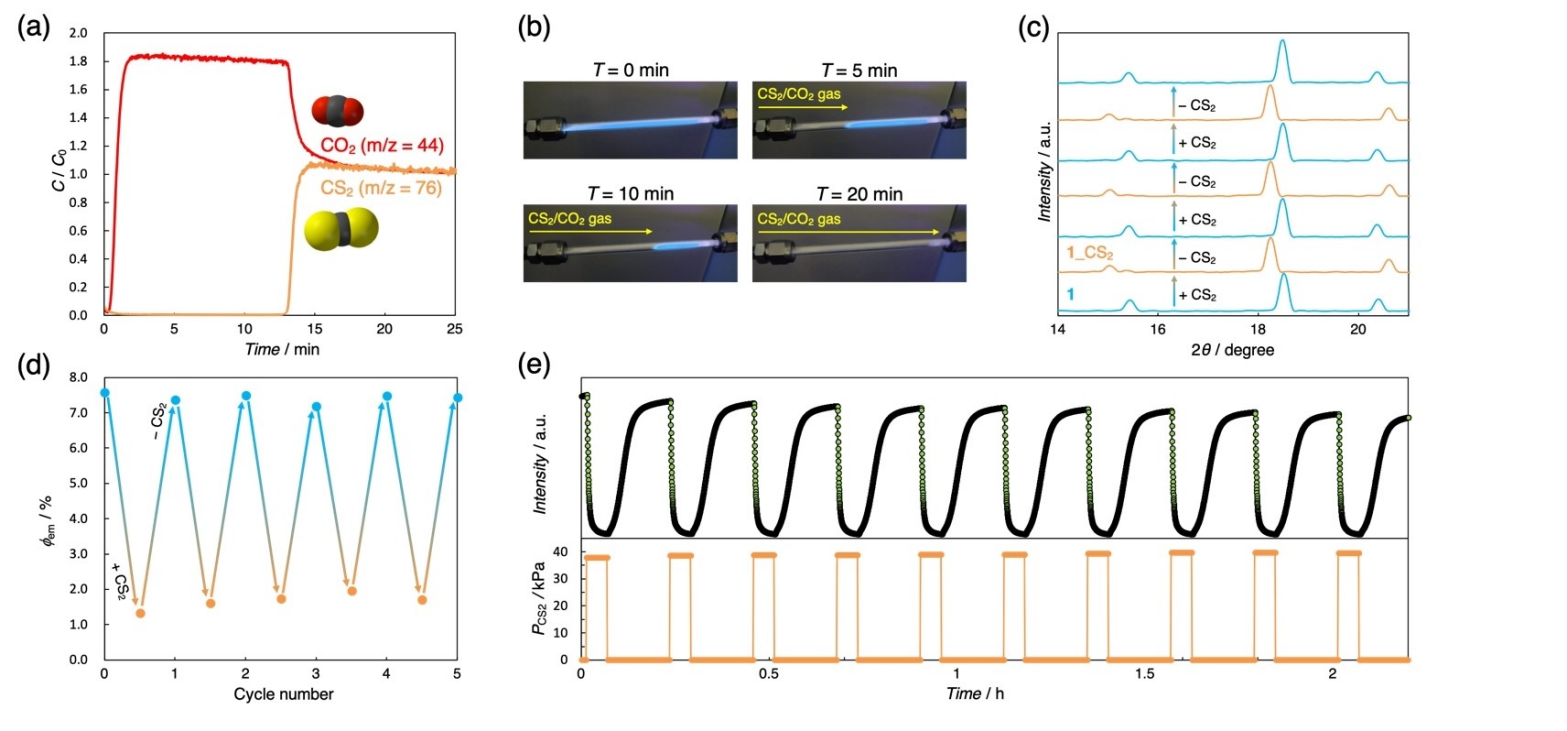
(a) Column breakthrough curve using CS_2_/CO_2_ (*P*/*P*, 21/79) gaseous mixture with 3 mL/min flow rate at RT, where *C*=concentration at a given time, *C*_0_=concentration after equilibrium is reached. (b) Photographs of the powder sample of **1** during the breakthrough experiment under UV light (*λ*_ex_=365 nm). CS_2_‐dependent reversible variations of (c) PXRD patterns and (d) *Φ*_em_ values at RT (λ_ex_=365 nm). (d) Time‐course measurements of the luminescence intensity (green circle) while the CS_2_ pressure (orange line) alternated between < 0.1 and 40 kPa for 10 cycles at RT.

Finally, we demonstrated the selectivity and reversibility of **1** for CS_2_ because ideal adsorbents for practical applications require favorable energy‐efficient regeneration and recyclability. The CS_2_‐selectivity was elucidated by breakthrough measurements. A gaseous mixture of CS_2_/CO_2_ (*P*/*P*, 21/79) was passed through a column cell filled with the powder samples of **1**. The breakthrough profile in Figure 4(a) exhibits a one‐step CS_2_ separation performance at RT, suggesting complete CS_2_‐selectivity. Importantly, continuous luminescence quenching was observed during the breakthrough test (Figure 4(b)), whose trend was consistent with the results of *in situ* emission spectroscopy under CS_2_ vapor (Figures 2(b) and (c)). Thus, these results mean “visualization of selective adsorption process for toxic gas in real‐time at RT”. Furthermore, *in situ* PXRD patterns displayed reversible conversions upon CS_2_ injection/ejection cycles (Figure 4(c)), indicating the structural stability of **1** for CS_2_ (The detailed measurement setup is described in SI). Compound **1** was reproduced by *in situ* vacuum treatment without heating between cycles. Moreover, the reversible response was also confirmed by PL spectroscopy. The *ex situ* PL spectra measured at RT have reversibly switched between *Φ*_em_=7.56 % (**1**) and *Φ*_em_=1.31 %(**1**_**CS_2_**) for at least five cycles (Figure 4(d)). To top it off, the switching of luminescence intensity between the guest‐free **1** phase under vacuum and **1**_**CS_2_** phase via dosing CS_2_ exhibits a rapid and reversible process (Figure 4(e)), highlighting the sustained recyclability of **1** for CS_2_ and demonstrating the potential character combining exceptional adsorption and sensing abilities toward toxic chemicals.

## Conclusion

In conclusion, we have described a novel multifunctional MOF of {Zn^II^(pz)[Au^I^(CN)_2_]_2_} (pz=pyrazine; **1**) which exhibits extremely specific adsorption and luminescent detection of harmful CS_2_ within an ultrafast response time of 10 s. The luminescence quantum efficiency of **1** drastically and reversibly varied with the CS_2_ adsorption/desorption processes, whose structural transformation accompanied by effective host−guest interactions could be found through *ex situ* single crystal X‐ray diffraction techniques. The significant decrease in the luminescent sites (that is, Au⋅⋅⋅Au interactions) caused by CS_2_‐induced structural conversion and manipulation of the energetic relaxation at the excited states provided the resultant luminescence changes with favorable visibility. Moreover, the steady‐state and time‐resolved photoluminescence systems under a gas atmosphere constructed in this study can serve as a new direction for investigating the intrinsic nature of gas‐responsive luminescent compounds. Finally, we wish to highlight that the concept of using luminescent centers in the frameworks as interaction sites for guest molecules would lead to novel insights into the rational design of highly gas‐sensing materials.

## Conflict of Interests

The authors declare no conflict of interest.

## Supporting information

As a service to our authors and readers, this journal provides supporting information supplied by the authors. Such materials are peer reviewed and may be re‐organized for online delivery, but are not copy‐edited or typeset. Technical support issues arising from supporting information (other than missing files) should be addressed to the authors.

Supporting Information
