# Supplementary material for: Ultrafast Luminescence Detection with Selective Adsorption of Carbon Disulfide in a Gold(I) Metal−Organic Framework
Source: Angew Chem Int Ed Engl. 2024 Dec 17;64(5):e202413830. doi: 10.1002/anie.202413830 (PMC11773104; doi:10.1002/anie.202413830)
Supplement: Supplementary file 1 — Supporting Information [file ANIE-64-e202413830-s001.pdf]

Angewandte Chemie
Eine Zeitschrift der Gesellschaft Deutscher Chemiker

Supporting Information

Ultrafast Luminescence Detection with Selective Adsorption of Carbon Disulfide in a Gold(I) Metal – Organic Framework

H. Yoshino, M. Saigo, T. Ehara, K. Miyata, K. Onda, J. Pirillo, Y. Hijikata, S. Takaishi, W. Kosaka, K.-i. Otake, S. Kitagawa, H. Miyasaka**

Supporting Information
©Wiley-VCH 2021
69451 Weinheim, Germany

Ultrafast Luminescence Detection with Selective Adsorption of Carbon Disulfide in a Gold(I) Metal–Organic Framework

Haruka Yoshino,* Masaki Saigo, Takumi Ehara, Kiyoshi Miyata, Ken Onda, Jenny Pirillo, Yuh Hijikata, Shinya Takaishi, Wataru Kosaka, Keni-ichi Otake, Susumu Kitagawa, and Hitoshi Miyasaka*

Abstract: Although a widely used and important industrial chemical, carbon disulfide (CS_2) poses a number of hazards due to its volatility and toxicity. As such, the development of multifunctional materials for the selective capture and easy recognition of CS_2 is one of the crucial issues. Herein, we demonstrate completely selective CS_2 adsorption among trials involving H_2O, alcohols, volatile organic compounds (including thiol derivatives), N_2, H_2, O_2, CH_4, CO, NO, and CO_2. We also showcase its fine detection using remarkable luminescent response in a gold(I)-based metal–organic framework (MOF) of $\{Zn^{II}(pz)[Au^{I}(CN)_2]_2\}$ (pz = pyrazine; **1**) with a two-fold interpenetration network. *Ex situ* single crystal X-ray diffraction for **1** and CS_2-accommodated **1** suggested that the Au···Au atoms are not only luminescent centers but also act as interaction sites for CS_2 modulating the Au···Au contacts. These experiments revealed the specificity of CS_2 and how changes in the CS_2-induced structure. Based on the obtained structural transformation, **1** exhibited a sensitive detecting ability for CS_2 with an ultrafast response time of less than 10 s. Moreover, *ex situ* time-resolved photoluminescence analyses developed in this work implied that CS_2 varied the energetic relaxation at the excited states related to the luminescent efficiency of the resultant MOF system.

DOI: 10.1002/anie.2021XXXXX

Table of Contents

Experimental Section ···················· 3-6
Table S1. Crystallographic data of **1** and **1_CS$_2$** ···················· 7
Fig. S1. Crystal views, crystal structure, and SEM image ···················· 8
Fig. S2, 3. IR and solid-state UV-vis reflectance spectra ···················· 9
Fig. S4, 5. TGA and emission decay curves ···················· 10
Fig. S6, 7. PXRD patterns and Raman spectra ···················· 11
Table S2. Photophysical properties of **1** and **1_CS$_2$** ···················· 12
Fig. S8. Calculated MOs of **1** and **1_CS$_2$** by DFT method ···················· 13
Table S3. Physical parameters of selected gas and vapor guests ···················· 14
Table S4. Cartesian coordinates of model for **1** and **1_CS$_2$** ···················· 15-19
Fig. S9. VT-CS$_2$ adsorption, adsorption heat of CS$_2$ ···················· 20
Table S5. Comparison of emission maximum energy from TR-PL results ···················· 21
Fig. S10. PXRD patterns under various solvent vapors ···················· 22
Fig. S11. PXRD patterns after heating and vacuum treatment ···················· 23
Fig. S12. *Ex situ* PL spectra of **1** under CS$_2$ at low concentration ···················· 24
Fig. S13. Comparison of thermal stability of **1_CS$_2$** after pz rotation ···················· 25
Fig. S14. 2D-plots of *ex situ* TR-PL spectra ···················· 26
References in SI ···················· 27

Experimental Procedures

Materials

Warning: Cyanide compounds are potentially toxic and should be handled with care and disposed of properly according to local regulations. Even if some cyanide salts have low solubility and might not create significant risks in themselves, they should still be handled with precaution, and their decomposition in strong acids should be avoided.

All chemicals were purchased from commercial sources and used without further purification. $Zn^{II}(pz)[Au^{I}(CN)_2]_2$ (**1**; pz = pyrazine) was prepared using the following steps.

Single crystals of **1** were prepared using the slow-diffusion method in a straight tube. A solution of $K[Au(CN)_2]$ (6.05 mg, 0.021 mmol) and pyrazine (1.68 mg, 0.0315 mmol) in H_2O (1.5 mL) was added to a solution of $ZnCl_2$ (2.86 mg, 0.021 mmol) in H_2O (1.5 mL) at room temperature. The solution was allowed to stand for several days and the colorless crystals were obtained. Elemental analysis (%): calculated. for $C_8H_4N_6ZnAu_2$: C 14.93, H 0.63, N 13.06; found: C 15.04, H 0.66, N 13.12. Powder samples of **1** for physical measurements were prepared for characterization by mixing the same components in H_2O. Yield 70.2%. Elemental analysis (%): calculated. for $C_8H_4N_6ZnAu_2$: C 14.93, H 0.63, N 13.06; found: C 15.03, H 0.69, N 13.15. Single crystals of carbon disulfide adsorbed state (**1_CS$_2$**) were prepared via a vapor diffusion method. The crystals of **1** were placed under CS_2 vapor for 1 h.

Physical Measurements

Elemental analysis of carbon, hydrogen and nitrogen for $Zn^{II}(pz)[Au^{I}(CN)_2]_2$ (**1**; pz = pyrazine) was conducted at the Division of the Graduate School of Science, Tohoku University. Infrared (IR) spectra were recorded using a JASCO FT/IR-4200 spectrophotometer with an ATR accessory in the range of 650–4000 cm^{-1} at room temperature (RT). Scanning electron microscopy (SEM) was conducted using a HITACHI S-3400N at the Collaborative Research and Development Center for Advanced Materials (CRDAM), Tohoku University (electron high tension (EHT) value:15.00 keV). Raman spectra were measured using a JASCO NRS-4500 Raman microscope at RT. The sample was inspected using an objective lens (Olympus MPLN20x), irradiating with a 532 nm Raman excitation laser. Powder X-ray diffraction (PXRD) measurements at RT were performed using a Rigaku Ultima IV spectrometer with graphite-monochromated Cu Kα radiation (λ = 1.5418 Å) and a D/teX Ultra detector. Powder samples of **1** were put into a silica glass capillary with an inner diameter of 0.5 mm. For the *in situ* PXRD measurements, the glass capillary was connected to stainless-steel lines that possessed a valve to dose and remove gas, which was connected to a pressure-handling system (BELSORP-MAX, Microtrac BEL Corp.) and home-made CS_2 injection system. Thermogravimetric analysis (TGA) was performed at a heating rate of 5 °C min^{-1} using a Shimadzu DTG-60H apparatus under a nitrogen atmosphere. Solid-state UV-vis reflectance spectra were collected on a JASCO V-770 spectrometer with an optical fibre integrating sphere system (HISV-729). Adsorption and desorption isotherms were acquired using BELSORP-MAX volumetric adsorption equipment (Microtrac BEL Corp.). The samples were activated by heating at 370 K for 6 h before the measurements. The adsorption enthalpy (ΔH_{ads}) for CS_2 was determined using the Clausius-Clapeyron equation with three temperatures (288 K, 298 K, and 308 K).[1,2] The breakthrough experiments were carried out using BELcat-II (Microtrac BEL Corp.). The outlet effluent from the column was continuously monitored using a quadrupole-type mass spectrometer (BEL-Mass, Microtrac BEL Corp.). The sample (*c.a.* 0.55 g) was packed into the column and activated before the breakthrough tests. The CO_2 and CS_2 (79%/21%) separation experiments were carried out at 283 K with a total gas flux rate of 3 mL/min. Emission and excitation spectra at RT were obtained using a JASCO FP-8550 spectrofluorometer with a dual-branch optical fiber and cryostat system (RC102, CRYO Industries). For the *in situ* photoluminescence measurement, the cryostat system was connected to stainless-steel lines that possessed a valve to dose and remove gas, which was connected to a pressure-handling system (BELSORP-MAX, Microtrac BEL Corp.) and home-made CS_2 injection system. Note that the response speed of **1** to CS_2 slightly varies depending on the presence or absence of a sample window in the cryostat (the sample window was used only in Fig.2(c) for the time course luminescent measurement).

The absolute emission quantum yield was measured using an absolute emission quantum yield measurement system (Hamamatsu Photonics C9920-02) compozed of an integrating sphere, a multichannel photodetector (Hamamatsu Photonics PMA-12), and a 150 W CW xenon lamp as an excitation light source (excitation wavelength = 365 nm) at RT.

The emission quantum yield (Φ) was calculated with the following equation:

$$\phi = \frac{\int I_{em}\, d\lambda}{\int (I_{ex}^{before} - I_{ex}^{after})\, d\lambda}$$

, where I_{em} is the number of photons, I^{before}_{ex} is the number of photons from the excitation light that is not absorbed, and I^{after}_{ex} is the number of photons from the excitation light that is absorbed.

Time-resolved photoluminescent (TR-PL) spectra including photoluminescence lifetimes (τ) were measured using a streak camera (Hamamatsu C4780) coupled to a polychromator (Acton Research Corporation, SpectraPro-150, spectral resolution: ~10 nm). The detection system was synchronized with a femtosecond Ti: sapphire chirped-pulse amplifier (Spectra-Physics, Spitfire Ace, pulse duration of 120 fs, central wavelength of 800 nm, and repetition rate of 1 kHz). The output of the amplifier was led to an optical parametric amplifier (Light conversion, TOPAS-prime), and the fourth harmonic (365 nm) of the

signal pulse (1460 nm) was used as the pump pulse. The polarization angles of the light for pumping/detection were set to the magic angle (54.7 °) to avoid distortion of the temporal profiles from the molecular orientation.[3] The excitation energy was maintained at less than 0.29 mJ/cm^2. In the measurements, the sample was packed into a home-made quartz cell with a quick seal (Microtrac BEL Corp.) attachable on top of the cell. The quartz cell was connected to stainless-steel lines that possessed a valve to dose and remove CS_2 gas, which was connected to a pressure-handling system (BELSORP-MAX, Microtrac BEL Corp.). The TR-PL measurements were collected after the sealing of the system.

The emission decay curves were fitted using a convolution of a Gaussian function, which is the instrument response function (IRF), and triple exponential functions. The Gaussian function is represented as:

$$f_{Gauss}(t) = \exp\left(-\frac{4\ln(2)\,(t-t_0)^2}{\mathrm{FWHM}^2}\right)$$

where t_0 is the peak position, FWHM is the full width at half maximum. The exponential function is represented as:

$$g_{Exp}(t) = \frac{1}{\tau}\exp\left(-\frac{t}{\tau}\right)$$

where τ represents the time constant. The convolution of two functions f(t) and g(t) is defined as:

$$(f * g)(t) = \int_{-\infty}^{\infty} f_{\mathrm{Gauss}}(\tau)\, g_{Exp}(t-\tau)\, d\tau$$

In order to mathematically express the convolution, the normal cumulative distribution function (ncdf) was introduced. The ncdf is defined as:

$$\mathrm{ncdf}(t) = \begin{cases} \dfrac{1-\mathrm{gammainc}(0.5, 0.5t^2)}{2} & if\ t < 0 \\ \dfrac{1+\mathrm{gammainc}(0.5, 0.5t^2)}{2} & otherwise \end{cases}$$

where the function gammainc represents the incomplete gamma function. Utilizing the ncdf, we formulate the combined exponential-Gaussian (ExpGauss) function, which is given by:

$$f_{\mathrm{ExpGauss}}(t, r, s) = r\exp(-rt + \frac{s^2r^2}{2})\mathrm{ncdf}(\frac{t}{s} - sr)$$

where r is the decay rate of the exponential function, s is the standard deviation of the Gaussian function. Therefore, the convolution of the Gaussian and triple exponential functions is:

$$\begin{aligned} &\mathrm{conv\_}GaussExp3(t, y0, t_0, \mathrm{FWHM}, \mathrm{amp}_1, \tau_1, \mathrm{amp}_2, \tau_2, \mathrm{amp}_3, \tau_3) \\ &= y0 \\ &+ \mathrm{amp}_1\tau_1 f_{ExpGauss}\left(t - t_0, \frac{1}{\tau_1}, \frac{\mathrm{FWHM}}{2\sqrt{2\ln(2)}}\right) \\ &+ \mathrm{amp}_2\tau_2 f_{ExpGauss}\left(t - t_0, \frac{1}{\tau_2}, \frac{\mathrm{FWHM}}{2\sqrt{2\ln(2)}}\right) \\ &+ \mathrm{amp}_3\tau_3 f_{ExpGauss}\left(t - t_0, \frac{1}{\tau_3}, \frac{\mathrm{FWHM}}{2\sqrt{2\ln(2)}}\right) \end{aligned}$$

Following this formulation, the average time constant, τ_{ave}, can be defined as a weighted average of the individual time constants τ_i. where each time constant is weighted by its respective amplitude, amp$_i$. τ_{ave} is expressed as:

$$\tau_{\mathrm{ave}} = \frac{\mathrm{amp}_1\tau_1 + \mathrm{amp}_2\tau_2 + \mathrm{amp}_3\tau_3}{\mathrm{amp}_1 + \mathrm{amp}_2 + \mathrm{amp}_3}$$

Single-crystal X-ray diffraction

Single crystals of **1** were prepared using the slow-diffusion method in a straight tube. A solution of K[$Au(CN)_2$] (6.05 mg, 0.021 mmol) and pyrazine (1.68 mg, 0.0315 mmol) in H_2O (1.5 mL) was added to a solution of single-crystal X-ray data for **1** and **1_CS₂** were recorded on a Rigaku XtaLAB AFC10 (RCD3) diffractometer with a monochromated Mo Kα radiation (λ = 0.71073 Å) equipped with Hybrid Pixel Array Detector. A single crystal of **1** was mounted on a polymer film with liquid paraffin and the temperature was controlled by a nitrogen flow using a Rigaku GN_2 apparatus. The single crystal of **1_CS_2** was obtained by a vapor diffusion method. A single crystal of **1** was mounted on the inner wall of the silica glass capillary with a minimum amount of silicon grease. After CS_2 vapor diffusion ($P/P_0 < 1$), the capillary was sealed off and used for the measurement. Data integration and reduction were undertaken with CrysAlisPro.[4] The structure was solved with the SHELXT structure solution program using direct methods and refined with the SHELXL refinement package using least squares minimization using Olex2 crystallography software.[5,6] Hydrogen atoms were included in idealized positions and refined using a riding model. The relevant crystal data collection and refinement data for the crystal structure of **1** are summarized in **Table S1**. CCDC 2368686 (**1**), 2368688, 2368689 (**1_CS_2**).

Computational analysis

The model structures of **1** and **1_CS_2** at ground (S_0) state were optimized under periodic boundary condition (PBC) using Crystal17 program,[7,8] referring to the crystal structure of **1** and **1_CS_2** at 298 K. The spin states of all Au atoms in the unit cell were fixed at S_0 state. We applied pob-TZVP-rev2 basis set [9] for C, H, N, and Zn, pob-TZVP-rev2 with an effective core potential (ECP) [10] for Au. PBE functional [11] with Grimme D3 type dispersion correction [12] were employed. The cell parameters were fixed during the optimization while maintaining *Fmmm* (**1**) and *Pmma* (**1_CS_2**) symmetry. The shrinking parameters were set to 2 and 2. After optimizing **1** and **1_CS_2** under PBC, using the optimized crystal structures, the [$Au_2(CN)_{16}(pz)_8$] unit was modelled, where the 12 CN^- ligands coordinating Zn ions were terminated by 0.5 electron charge. The investigation of the MOs was carried out with M06 functional [13] and 6-311G(d,p) for C, H, and N and LANL2DZ with ECP for Au and Zn were employed. All model calculations were performed using Gaussian 16 Rev. C.01.[14]

Table S1. Crystallographic data and refinement parameters of **1** and **1_CS$_2$**.

	**1**	**1_CS$_2$**	**1_CS$_2$**
T / K	298 K	298 K	150 K
CCDC	2368686	2368688	2368689
Formula	$C_8H_4N_6ZnAu_2$	$C_{8.5}H_4N_6SZnAu_2$	$C_{8.5}H_4N_6SZnAu_2$
Formula weight	643.47	681.54	681.54
Crystal system	Orthorhombic	Orthorhombic	Orthorhombic
Space group	*Fmmm*	*Pmma*	*Pmma*
a / Å	7.0871(7)	17.1540(9)	17.2486(13)
b / Å	17.3352(14)	11.6472(7)	11.5189(10)
c / Å	11.3819(10)	7.0935(4)	7.0756(5)
α / °	90	90	90
β / °	90	90	90
γ / °	90	90	90
V / Å^3	1398.3(2)	1417.25(14)	1405.82(19)
Z	4	4	4
$R_1{}^a$	0.0146	0.0310	0.0253
$wR_2{}^b$	0.0307	0.0721	0.0614
Goodness of Fit	1.009	1.130	1.064

^{a} $R_1 = R = \Sigma||F_o|-|F_c||/\Sigma|F_o|$. ^{b} $wR_2 = [\Sigma w(F_o{}^2-F_c{}^2)^2/\Sigma w(F_o{}^2)^2]^{1/2}$

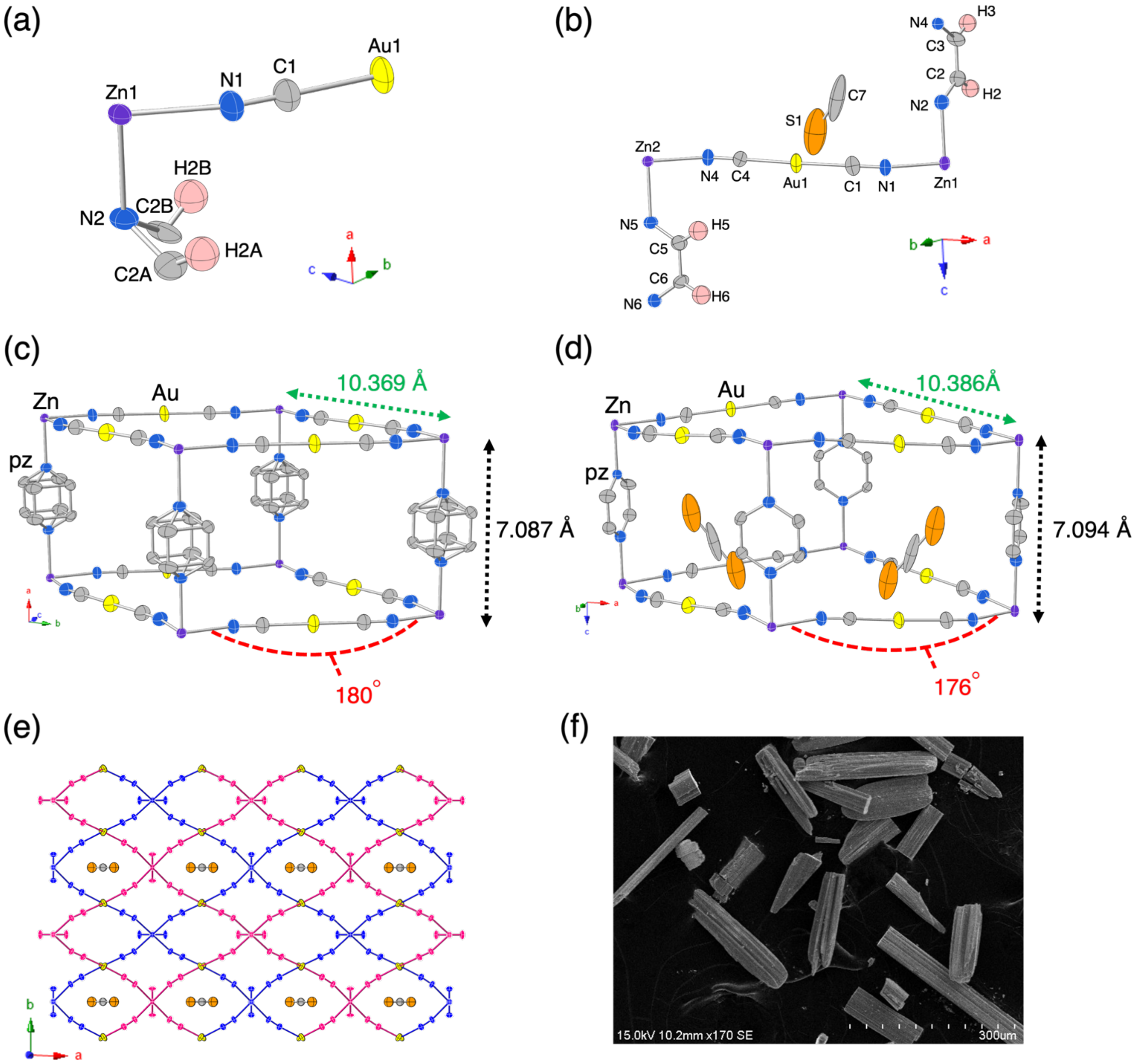

Fig. S1. ORTEP drawings of the asymmetric unit for (a) **1** and (b) **1_CS₂** at 298 K. Atomic code: Zn, purple; Au, yellow; C, grey; N, blue; S, orange; H, light pink, respectively. Thermal ellipsoids are shown at the 50% probability level. The sub-lattice within the framework of (c) **1** and (d) **1_CS₂** at 298 K. (e) Packing structure of **1_CS₂** viewed along the *c*-axis. Au atoms are represented by yellow spheres and deep blue and pink highlight the two-fold interpenetration networks. H atoms are omitted for clarity. (f) A scanning electron microscopy (SEM) image of **1** (Electron high tension (EHT) value: 15.00 keV)

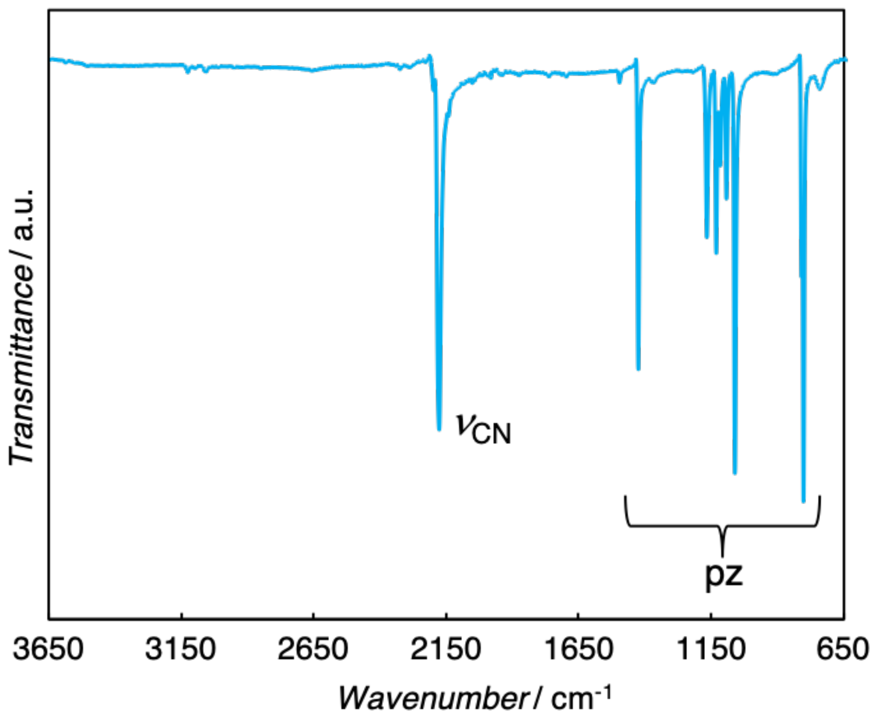

Fig. S2. IR spectra of **1** at RT.

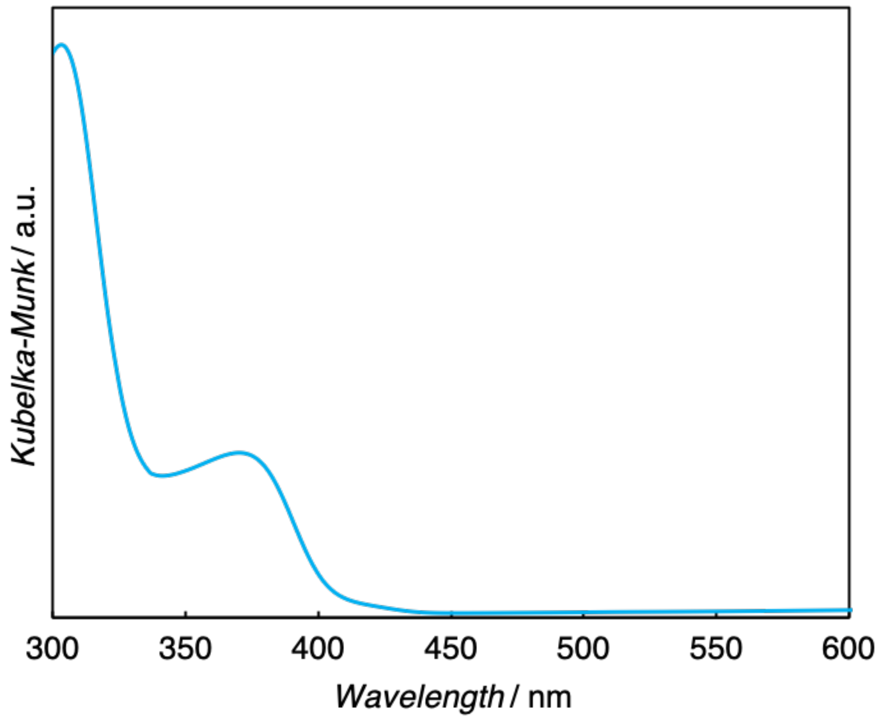

Fig. S3. Solid-state UV-vis reflectance spectra of **1** at RT.

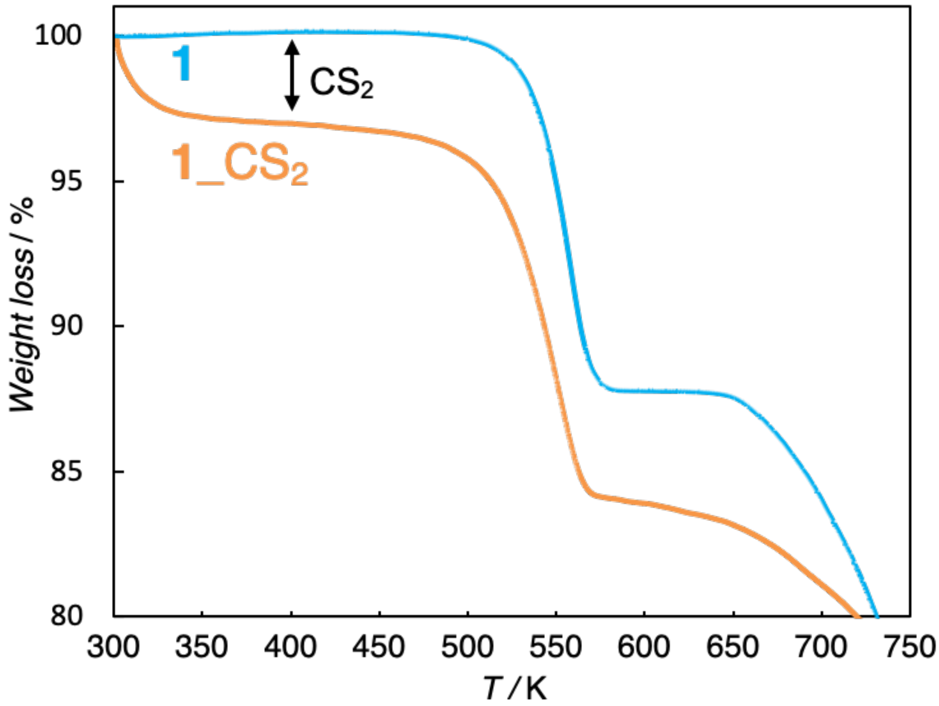

Fig. S4. TGA curves of **1** (light blue) and **1_CS₂** (orange) (Heating rate: 5 °C/min under N_2 flow).

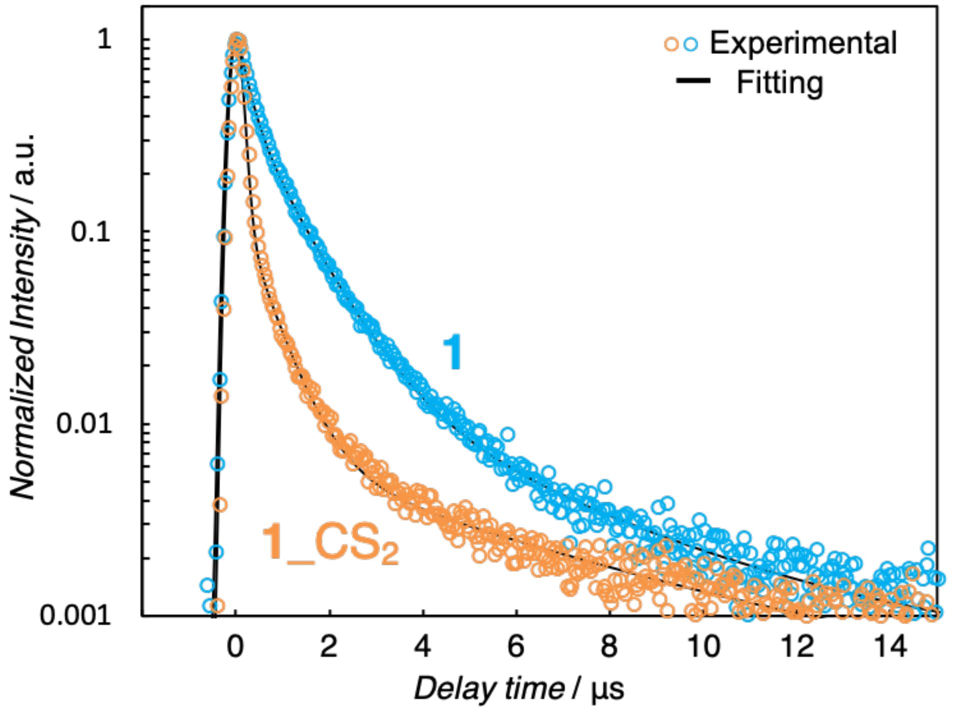

Fig. S5. Emission decay curves of **1** (light blue) and **1_CS₂** (orange) in the range of 400-550 nm at RT (λ_{ex} = 365 nm).

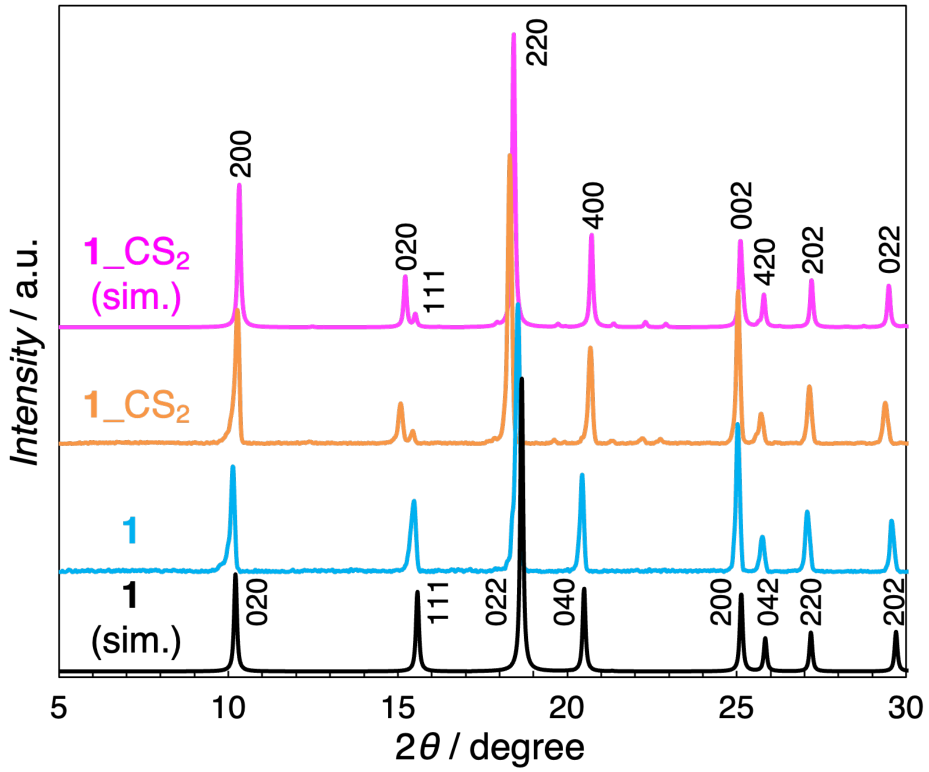

Fig. S6. PXRD patterns of **1** (light blue), **1_CS₂** (orange), and their simulated patterns (black, pink) at RT (λ = 1.5418 Å).

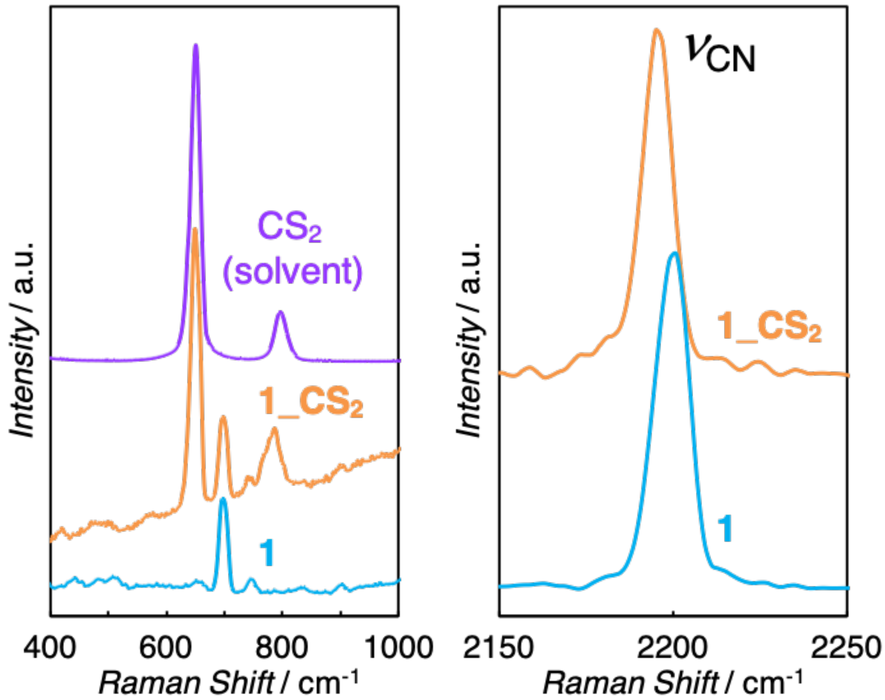

Fig. S7. Raman spectra of **1** (light blue), **1_CS₂** (orange), and CS_2 solvent (purple) at RT.

Table S2. Photophysical properties of **1** and **1_CS_2**.

	**1**	**1_CS_2**
$\Phi_{em}{}^{a}$ / %	7.41	1.66
$\tau_{ave.}{}^{b}$ / µs	0.482	0.101
τ_1 / µs	0.224170066	0.074503047
(A_1 / -)	1.156858117	3.890431901
τ_2 / µs	0.902757771	0.581365238
(A_2 / -)	0.531212808	0.145631239
τ_3 / µs	3.783327502	5.1338944
(A_3 / -)	0.022541169	0.006735449
$k_r{}^{d}$ / s^{-1}	1.54×10^5	1.64×10^5
$k_{nr}{}^{e}$ / s^{-1}	1.92×10^6	9.72×10^6

[a] Average photoluminescence quantum yields at RT shown in Fig. 4(d). [b] $\tau_{ave} = (\Sigma A_n \tau_n)/(\Sigma A_n)$, where A_n and τ_{ave} represent pre-exponentials and the weighted average photoluminescence lifetime, respectively. [c] Radiative decay rate constants (k_r) were estimated using the equation Φ_{em}/τ_{ave}. [d] Nonradiative decay rate constants (k_{nr}) were estimated from the equation $k_r\ (1 - \Phi_{em})/\Phi_{em}$.

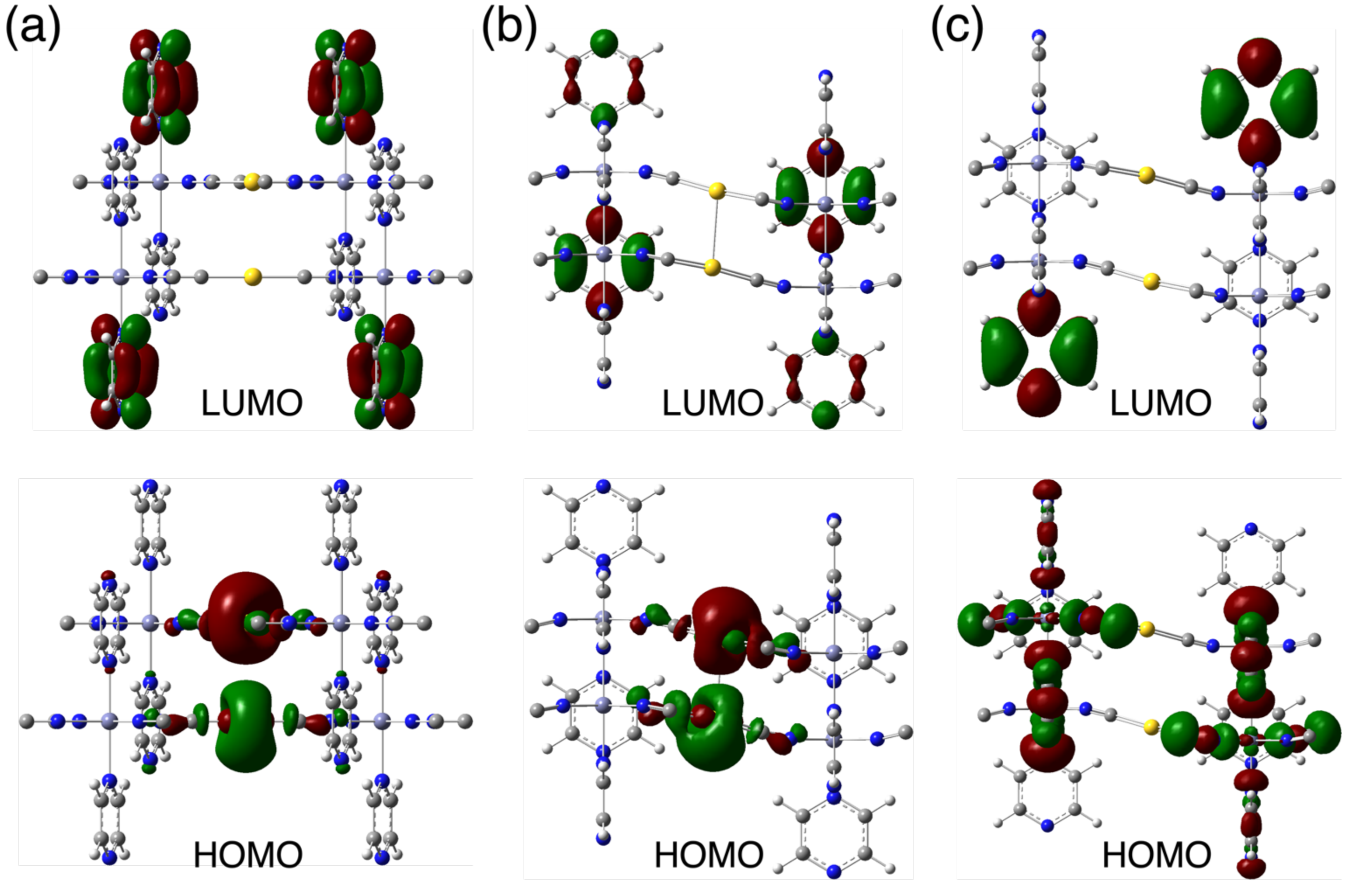

Fig. S8. Calculated the highest occupied molecular orbital (HOMO) and the lowest unoccupied MO (LUMO) of the model for (a) **1**, (b) site A structure of **1_CS₂**, and (c) site B structure of **1_CS₂** at the singlet (S_0) state by DFT calculations. Atomic code: Zn, orchid; Au, yellow; C, grey; N, blue; H, white, respectively.

Table S3. Physical parameters of selected gas and vapor guests.[15]

Guest	Kinetic diameter/ Å	V / cm^3 mol^{-1}
CS_2	4.483	173
CO_2	3.3	94.07
N_2	3.64–3.80	90.10
O_2	3.467	73.37
H_2	2.827–2.89	64.20
CH_4	3.758	98.60
CO	3.690	93.10
NO	3.492	58.00
H_2O	2.641	55.95
MeOH	3.626	118.00
EtOH	4.530	167.00

Table S4. Cartesian coordinates of model for **1** and **1_CS$_2$** (site A and B structures).

1

Au	0	0	-1.771775	N	-4.3338	-2.845476	-3.929762
Au	0	0	1.771775	C	6.89433	4.862225	-1.771774
C	4.333799	3.99731	-6.008923	C	-1.77327	4.862224	1.771777
C	4.3338	3.99731	-4.621725	C	1.77327	4.862224	-1.771777
H	4.3338	4.9224	-6.577532	C	4.3338	3.997308	1.078177
H	4.3338	4.9224	-4.053117	C	4.3338	3.997309	2.465375
C	4.3338	1.693642	-4.621726	H	4.333799	4.922399	0.509568
C	4.3338	-3.997309	-2.465375	H	4.3338	4.922398	3.033984
C	4.333799	1.693642	-6.008924	N	-2.801479	4.354802	1.771776
C	4.3338	-1.69364	-2.465374	N	5.86612	4.354803	-1.771774
H	4.3338	0.768552	-4.053118	N	2.801479	4.354802	-1.771776
H	4.3338	-4.922398	-3.033984	C	-6.89433	4.862225	1.771774
H	4.3338	0.768553	-6.577533	C	-4.3338	3.99731	4.621725
H	4.3338	-0.76855	-3.033983	C	-4.3338	3.997308	-1.078177
N	4.3338	-2.845474	-3.157339	H	-4.3338	4.9224	4.053117
N	4.333799	2.845477	-6.700888	H	-4.333799	4.922399	-0.509568
N	4.3338	2.845476	-3.929762	N	-5.86612	4.354803	1.771774
C	-4.3338	3.997309	-2.465375	C	-4.333799	3.99731	6.008923
H	-4.3338	4.922398	-3.033984	H	-4.3338	4.9224	6.577532
C	-4.3338	-1.693642	-4.621726	C	-1.77327	-0.828725	-1.771775
C	-4.3338	-3.99731	-4.621725	C	6.89433	0.828726	-1.771775
C	-4.333799	-3.99731	-6.008923	C	6.89433	-4.862225	1.771774
C	-4.3338	1.69364	-2.465374	C	-1.77327	0.828725	1.771775
C	-4.333799	-1.693642	-6.008924	C	1.77327	0.828725	-1.771775
H	-4.3338	-0.768552	-4.053118	C	1.77327	-4.862224	1.771777
H	-4.3338	-4.9224	-4.053117	C	-1.77327	-4.862224	-1.771777
H	-4.3338	-4.9224	-6.577532	C	6.89433	-0.828726	1.771775
H	-4.3338	0.76855	-3.033983	C	1.77327	-0.828725	1.771775
H	-4.3338	-0.768553	-6.577533	C	4.3338	-3.997308	-1.078177
N	-4.3338	2.845474	-3.157339	C	4.3338	1.69364	2.465374
N	-4.333799	-2.845477	-6.700888	C	4.3338	-3.99731	4.621725
C	4.3338	1.693641	1.078177	C	-4.3338	-3.997309	2.465375
C	4.3338	-1.693642	4.621726	C	-4.3338	-3.997308	1.078177
C	4.3338	-1.693641	-1.078177	C	-4.3338	1.693641	-1.078177
H	4.333799	-4.922399	-0.509568	C	-4.3338	1.693642	4.621726
H	4.3338	0.76855	3.033983	C	-4.3338	-1.693641	1.078177
H	4.3338	-4.9224	4.053117	H	-4.3338	-0.76855	3.033983
H	4.3338	0.768552	0.509568	H	-4.3338	-4.922398	3.033984
H	4.3338	-0.768552	4.053118	H	-4.333799	-4.922399	0.509568
H	4.3338	-0.768552	-0.509568	H	-4.3338	0.768552	-0.509568
N	5.86612	-4.354803	1.771774	H	-4.3338	0.768552	4.053118
N	5.866121	1.336148	-1.771775	H	-4.3338	-0.768552	0.509568
N	-2.801479	-4.354802	-1.771776	N	-5.866121	-1.336148	-1.771775
N	2.801479	-4.354802	1.771776	N	-5.86612	-4.354803	-1.771774
N	2.82766	1.349069	-1.771774	N	-5.866121	1.336148	1.771775
N	-2.82766	1.349069	1.771774	N	-4.3338	2.845476	3.929762
N	5.866121	-1.336148	1.771775	N	-4.3338	2.845475	-0.386213
N	-2.82766	-1.349069	-1.771774	N	-4.3338	-2.845475	0.386213
N	2.82766	-1.349069	1.771774	N	-4.3338	-2.845474	3.157339

N	4.3338	-2.845476	3.929762	Zn	-4.3338	2.845475	1.771774
N	4.3338	2.845475	0.386213	Zn	-4.3338	-2.845475	-1.771774
N	4.3338	-2.845475	-0.386213	C	4.333799	-3.99731	6.008923
N	4.3338	2.845474	3.157339	C	4.333799	-1.693642	6.008924
Zn	4.3338	-2.845475	1.771774	H	4.3338	-4.9224	6.577532
Zn	4.3338	2.845475	-1.771774	H	4.3338	-0.768553	6.577533
C	-6.89433	0.828726	1.771775	N	4.333799	-2.845477	6.700888
C	-6.89433	-0.828726	-1.771775	C	-4.333799	1.693642	6.008924
C	-6.89433	-4.862225	-1.771774	H	-4.3338	0.768553	6.577533
C	-4.3338	-1.69364	2.465374	N	-4.333799	2.845477	6.700888

site A structure of **1_CS_2**,

Au	0.101793	1.482378	-0.147111	C	-6.751357	-1.161437	5.132244
Au	-0.101793	-1.482378	-0.147111	C	4.2885	-3.265583	4.103638
C	1.623068	-1.967618	-4.782041	C	4.2885	-3.265583	1.789972
C	-1.623068	1.967618	-4.782041	C	-4.2885	3.265583	4.103638
C	-6.953932	1.967618	-4.782041	C	-4.2885	3.265583	1.789972
C	6.953932	-1.967618	-4.782041	C	4.2885	-1.874504	4.10495
N	2.677164	-2.219033	-4.319504	C	4.2885	-1.874504	1.78866
N	-2.677164	2.219033	-4.319504	C	-4.2885	1.874504	4.10495
N	-5.899836	2.219033	-4.319504	C	-4.2885	1.874504	1.78866
N	5.899836	-2.219033	-4.319504	C	6.953932	-1.967618	-0.971549
C	-4.2885	-3.827917	4.103638	C	-6.953932	1.967618	-0.971549
C	-4.2885	-3.827917	1.789972	C	-1.623068	1.967618	-0.971549
C	-4.2885	-5.218996	4.10495	C	1.623068	-1.967618	-0.971549
C	-4.2885	-5.218996	1.78866	C	3.132818	0.621796	-2.876795
C	3.132818	-6.471704	-2.876795	C	5.444182	0.621796	-2.876795
C	5.444182	-6.471704	-2.876795	C	-3.132818	-0.621796	-2.876795
C	3.132078	-5.082417	-2.876795	C	-5.444182	-0.621796	-2.876795
C	5.444922	-5.082417	-2.876795	C	3.132078	2.011083	-2.876795
H	4.2885	-3.844024	5.028043	C	5.444922	2.011083	-2.876795
H	4.2885	-3.844024	0.865567	C	-3.132078	-2.011083	-2.876795
H	-4.2885	-5.795928	5.030539	C	-5.444922	-2.011083	-2.876795
H	-4.2885	-5.795928	0.863071	H	4.2885	3.249476	5.028043
H	2.203243	-7.042867	-2.876795	H	4.2885	3.249476	0.865567
H	6.373757	-7.042867	-2.876795	H	-4.2885	-3.249476	5.028043
H	2.202632	-4.509107	-2.876795	H	-4.2885	-3.249476	0.865567
H	6.374368	-4.509107	-2.876795	H	4.2885	-1.297572	5.030539
N	4.2885	-3.955424	2.946805	H	4.2885	-1.297572	0.863071
N	-4.2885	-5.907728	2.946805	H	-4.2885	1.297572	5.030539
N	4.2885	-7.164298	-2.876795	H	-4.2885	1.297572	0.863071
N	4.2885	-4.391343	-2.876795	H	2.203243	0.050633	-2.876795
C	4.2885	3.827917	4.103638	H	6.373757	0.050633	-2.876795
C	4.2885	3.827917	1.789972	H	-2.203243	-0.050633	-2.876795
C	4.2885	5.218996	4.10495	H	-6.373757	-0.050633	-2.876795
C	4.2885	5.218996	1.78866	H	2.202632	2.584393	-2.876795
C	-3.132818	6.471704	-2.876795	H	6.374368	2.584393	-2.876795
C	-5.444182	6.471704	-2.876795	H	-2.202632	-2.584393	-2.876795

C	-3.132078	5.082417	-2.876795	H	-6.374368	-2.584393	-2.876795
C	-5.444922	5.082417	-2.876795	N	-2.787627	-0.987848	4.479133
H	-4.2885	3.844024	5.028043	N	-5.789373	-0.987848	1.414477
H	-4.2885	3.844024	0.865567	N	2.787627	0.987848	4.479133
H	4.2885	5.795928	5.030539	N	5.789373	0.987848	1.414477
H	4.2885	5.795928	0.863071	N	2.787627	0.987848	1.414477
H	-2.203243	7.042867	-2.876795	N	5.789373	0.987848	4.479133
H	-6.373757	7.042867	-2.876795	N	-2.787627	-0.987848	1.414477
H	-2.202632	4.509107	-2.876795	N	-5.789373	-0.987848	4.479133
H	-6.374368	4.509107	-2.876795	N	4.2885	3.138076	2.946805
N	-4.2885	3.955424	2.946805	N	-4.2885	-3.138076	2.946805
N	4.2885	5.907728	2.946805	N	4.2885	-1.185772	2.946805
N	-4.2885	7.164298	-2.876795	N	-4.2885	1.185772	2.946805
N	-4.2885	4.391343	-2.876795	N	5.899836	-2.219033	-1.434086
Au	0.101793	1.482378	-0.147111	N	-5.899836	2.219033	-1.434086
Au	-0.101793	-1.482378	-0.147111	N	-2.677164	2.219033	-1.434086
C	1.623068	-1.967618	-4.782041	N	2.677164	-2.219033	-1.434086
C	-1.623068	1.967618	-4.782041	N	4.2885	-0.070798	-2.876795
C	-6.953932	1.967618	-4.782041	N	-4.2885	0.070798	-2.876795
C	6.953932	-1.967618	-4.782041	N	4.2885	2.702157	-2.876795
N	2.677164	-2.219033	-4.319504	N	-4.2885	-2.702157	-2.876795
N	-2.677164	2.219033	-4.319504	Zn	4.2885	0.968438	2.946805
N	-5.899836	2.219033	-4.319504	Zn	-4.2885	-0.968438	2.946805
C	-1.825643	-1.161437	5.132244	Zn	4.2885	-2.243461	-2.876795
C	-6.751357	-1.161437	0.761366	Zn	-4.2885	2.243461	-2.876795
C	1.825643	1.161437	5.132244	C	-6.751357	-1.161437	5.132244
C	6.751357	1.161437	0.761366	C	4.2885	-3.265583	4.103638
C	1.825643	1.161437	0.761366	C	4.2885	-3.265583	1.789972
C	6.751357	1.161437	5.132244	C	-4.2885	3.265583	4.103638
C	-1.825643	-1.161437	0.761366	C	-4.2885	3.265583	1.789972

site B structure of **1_CS$_2$**,

Au	-0.101793	2.064372	-0.147111	N	2.677164	1.327717	-1.434086
Au	0.101793	-2.064372	-0.147111	N	4.2885	3.475952	-2.876795
C	-1.623068	-1.579132	-4.782041	N	-4.2885	3.617548	-2.876795
C	-6.953932	-1.579132	-4.782041	N	4.2885	6.248907	-2.876795
N	-2.677164	-1.327717	-4.319504	N	-4.2885	0.844593	-2.876795
N	-5.899836	-1.327717	-4.319504	Zn	-4.2885	2.578312	2.946805
C	1.623068	1.579132	-4.782041	Zn	4.2885	1.303289	-2.876795
C	6.953932	1.579132	-4.782041	H	-4.2885	7.390774	5.028043
N	2.677164	1.327717	-4.319504	H	-4.2885	7.390774	0.865567
N	5.899836	1.327717	-4.319504	N	-4.2885	7.502174	2.946805
H	4.2885	-7.390774	5.028043	C	1.825643	-2.385313	5.132244
H	4.2885	-7.390774	0.865567	C	6.751357	-2.385313	0.761366
N	4.2885	-7.502174	2.946805	C	1.825643	-2.385313	0.761366
C	-1.825643	2.385313	5.132244	C	6.751357	-2.385313	5.132244
C	-6.751357	2.385313	0.761366	C	4.2885	-6.812333	4.103638

C	-1.825643	2.385313	0.761366	C	4.2885	-6.812333	1.789972
C	-6.751357	2.385313	5.132244	C	-4.2885	-0.281167	4.103638
C	4.2885	0.281167	4.103638	C	-4.2885	-0.281167	1.789972
C	4.2885	0.281167	1.789972	C	4.2885	-5.421254	4.10495
C	-4.2885	6.812333	4.103638	C	4.2885	-5.421254	1.78866
C	-4.2885	6.812333	1.789972	C	-4.2885	-1.672246	4.10495
C	4.2885	1.672246	4.10495	C	-4.2885	-1.672246	1.78866
C	4.2885	1.672246	1.78866	C	-6.953932	-1.579132	-0.971549
C	-4.2885	5.421254	4.10495	C	-1.623068	-1.579132	-0.971549
C	-4.2885	5.421254	1.78866	C	3.132818	-2.924954	-2.876795
C	6.953932	1.579132	-0.971549	C	5.444182	-2.924954	-2.876795
C	1.623068	1.579132	-0.971549	C	-3.132818	-4.168546	-2.876795
C	3.132818	4.168546	-2.876795	C	-5.444182	-4.168546	-2.876795
C	5.444182	4.168546	-2.876795	C	3.132078	-1.535667	-2.876795
C	-3.132818	2.924954	-2.876795	C	5.444922	-1.535667	-2.876795
C	-5.444182	2.924954	-2.876795	C	-3.132078	-5.557833	-2.876795
C	3.132078	5.557833	-2.876795	C	-5.444922	-5.557833	-2.876795
C	5.444922	5.557833	-2.876795	H	4.2885	-0.297274	5.028043
C	-3.132078	1.535667	-2.876795	H	4.2885	-0.297274	0.865567
C	-5.444922	1.535667	-2.876795	H	4.2885	-4.844322	5.030539
H	-4.2885	0.297274	5.028043	H	4.2885	-4.844322	0.863071
H	-4.2885	0.297274	0.865567	H	-4.2885	-2.249178	5.030539
H	4.2885	2.249178	5.030539	H	-4.2885	-2.249178	0.863071
H	4.2885	2.249178	0.863071	H	2.203243	-3.496117	-2.876795
H	-4.2885	4.844322	5.030539	H	6.373757	-3.496117	-2.876795
H	-4.2885	4.844322	0.863071	H	-2.203243	-3.597383	-2.876795
H	2.203243	3.597383	-2.876795	H	-6.373757	-3.597383	-2.876795
H	6.373757	3.597383	-2.876795	H	2.202632	-0.962357	-2.876795
H	-2.203243	3.496117	-2.876795	H	6.374368	-0.962357	-2.876795
H	-6.373757	3.496117	-2.876795	H	-2.202632	-6.131143	-2.876795
H	2.202632	6.131143	-2.876795	H	-6.374368	-6.131143	-2.876795
H	6.374368	6.131143	-2.876795	N	2.787627	-2.558902	4.479133
H	-2.202632	0.962357	-2.876795	N	5.789373	-2.558902	1.414477
H	-6.374368	0.962357	-2.876795	N	2.787627	-2.558902	1.414477
N	-2.787627	2.558902	4.479133	N	5.789373	-2.558902	4.479133
N	-5.789373	2.558902	1.414477	N	4.2885	-0.408674	2.946805
N	-2.787627	2.558902	1.414477	N	4.2885	-4.732522	2.946805
N	-5.789373	2.558902	4.479133	N	-4.2885	-2.360978	2.946805
N	-4.2885	0.408674	2.946805	N	-5.899836	-1.327717	-1.434086
N	4.2885	2.360978	2.946805	N	-2.677164	-1.327717	-1.434086
N	-4.2885	4.732522	2.946805	N	4.2885	-3.617548	-2.876795
N	5.899836	1.327717	-1.434086	N	-4.2885	-3.475952	-2.876795
Au	-0.101793	2.064372	-0.147111	N	4.2885	-0.844593	-2.876795
Au	0.101793	-2.064372	-0.147111	N	-4.2885	-6.248907	-2.876795
C	-1.623068	-1.579132	-4.782041	Zn	4.2885	-2.578312	2.946805
C	-6.953932	-1.579132	-4.782041	Zn	-4.2885	-1.303289	-2.876795
N	-2.677164	-1.327717	-4.319504	N	2.677164	1.327717	-1.434086
N	-5.899836	-1.327717	-4.319504	N	4.2885	3.475952	-2.876795

C	1.825643	1.161437	0.761366	N	-4.2885	3.617548	-2.876795
C	6.751357	1.161437	5.132244	N	4.2885	6.248907	-2.876795
N	-1.825643	-1.161437	0.761366	N	-4.2885	0.844593	-2.876795

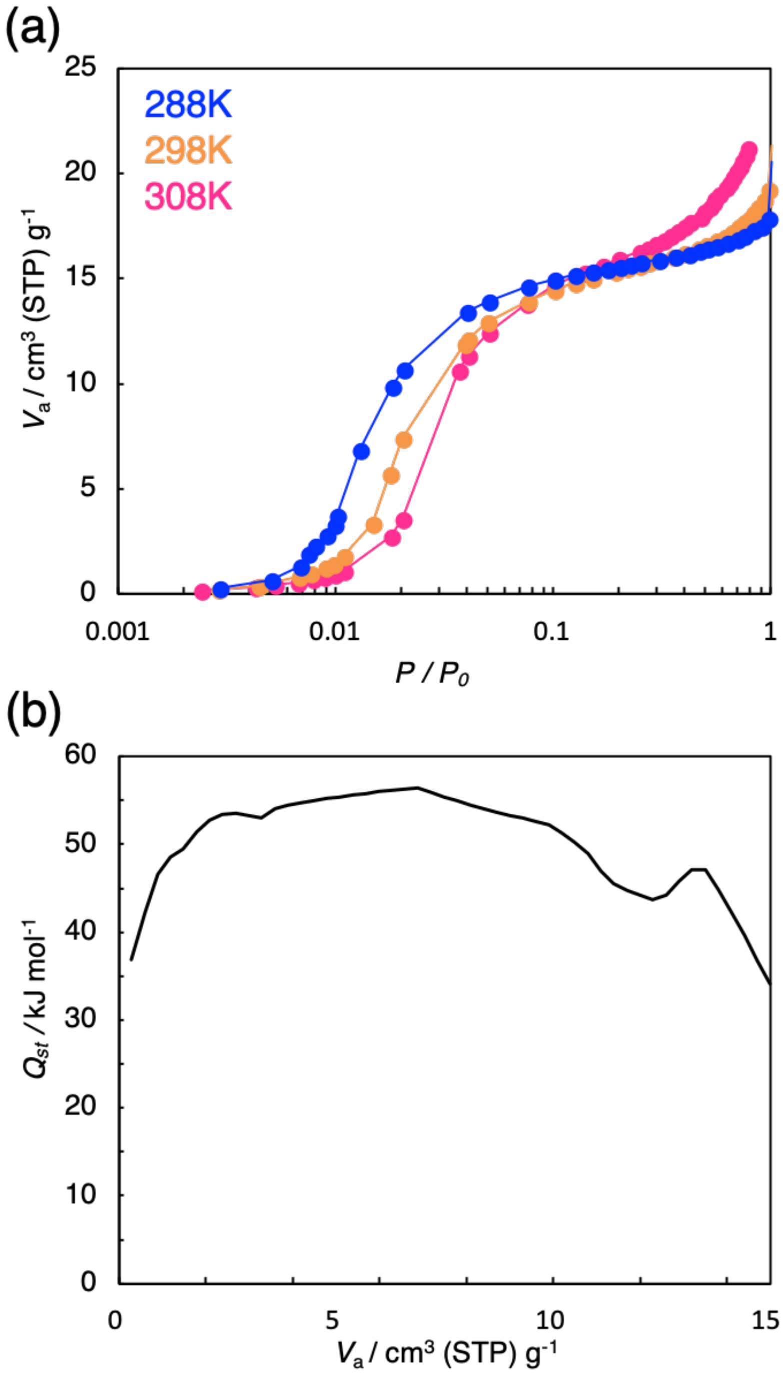

Fig. S9. (a) Variable-temperature CS_2-adsorption isotherms of **1** at 288 K (deep blue), 298 K (orange), and 308 K (deep pink) with log plots. Solid lines represent a guide for easier viewing. (b) Adsorption heat of CS_2 in **1** estimated by Clausius-Clapeyron equation.

Table S5. Comparison of emission maximum energy for **1** and **1_CS₂** within the initial state immediately after excitation ($E_{init.}$), the relaxed state ($E_{relax.}$), and the difference between the two energies ($\Delta E = E_{init.} - E_{relax.}$), obtained using *ex situ* TR-PL results.

	**1**	**1_CS$_2$**
$E_{init.}$[a] / eV	2.93	2.89
$E_{relax.}$[b] / eV	2.69	2.37
ΔE[c] / eV	0.24	0.52

[a] Emission maximum of the initial state immediately after excitation (~200 ps). [b] Emission maximum of the relaxed state (~10 μs). [c] Difference between $E_{init.}$ and $E_{relax.}$.

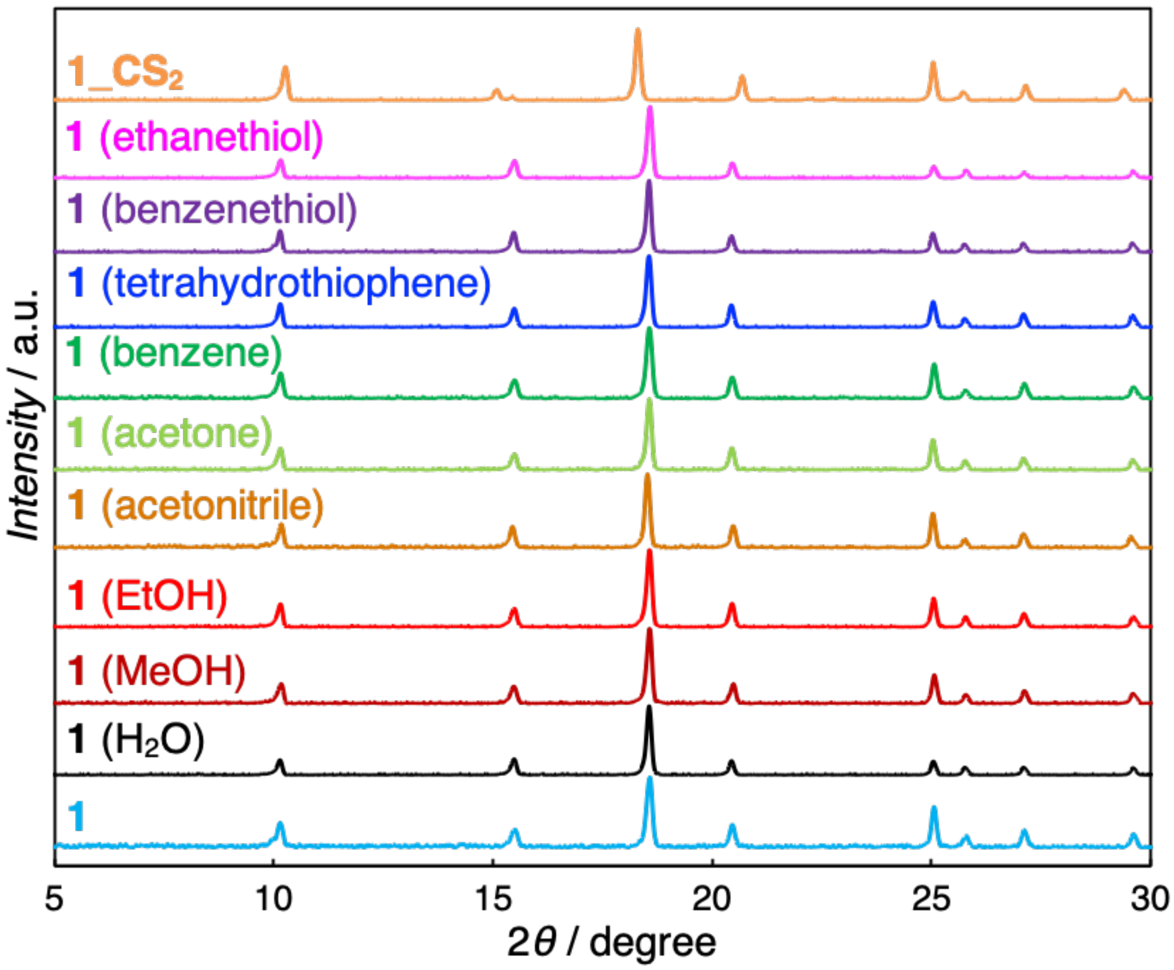

Fig. S10. PXRD patterns of **1** under vapor of H_2O (black), MeOH (brown), EtOH (red), acetonitrile (ocher), acetone (light green), benzene (green), tetrahydrothiophene (deep blue), benzenethiol (purple), ethanethiol (pink), and CS_2 (orange) at RT (λ = 1.5418 Å). Vapor diffusion treatments were carried out for 24 h.

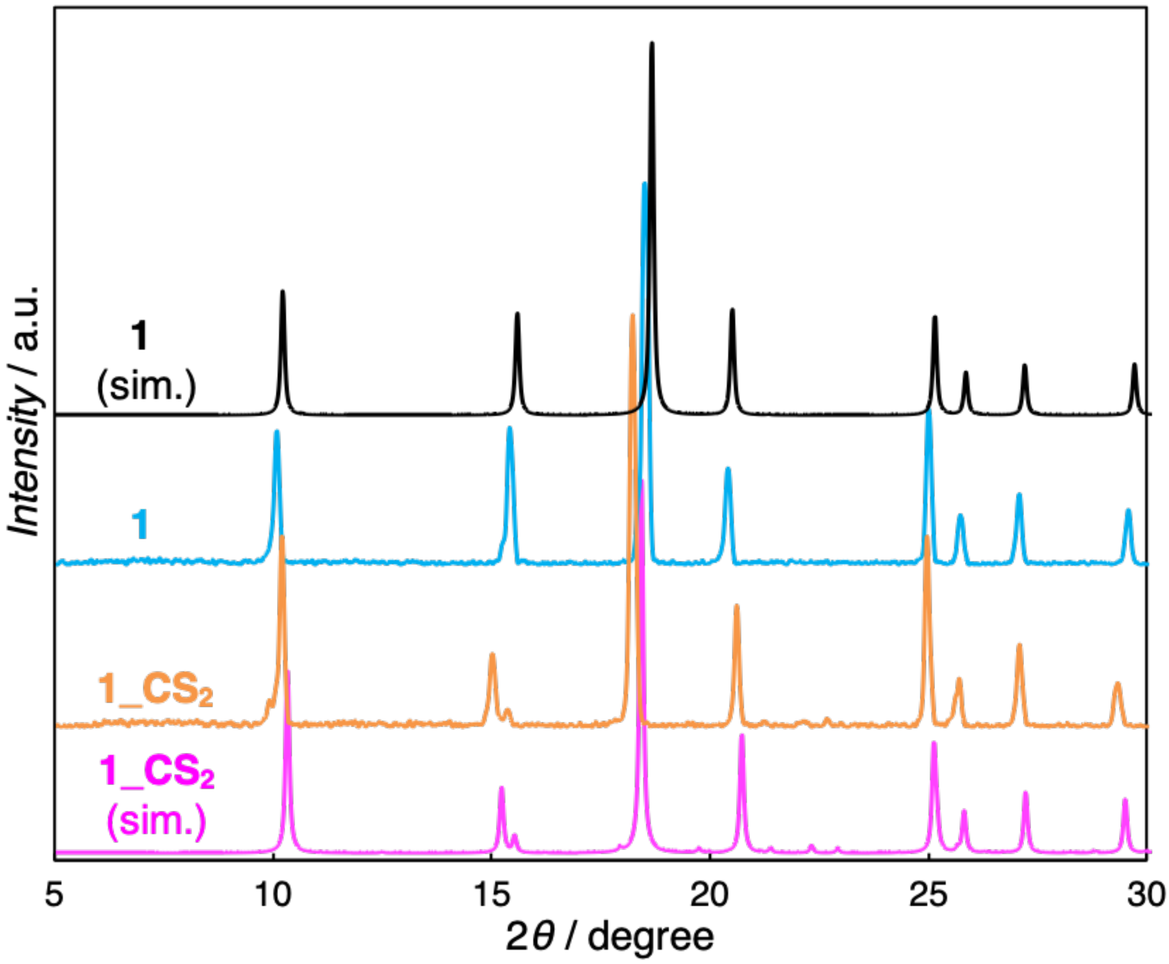

Fig. S11. PXRD patterns of **1_CS₂** (orange) and **1** (light blue) after heating and vacuum treatment, and their simulated patterns (pink, black) at RT (λ = 1.5418 Å). The heating and vacuum treatment of **1_CS₂** were carried out at 373 K for 1 minute.

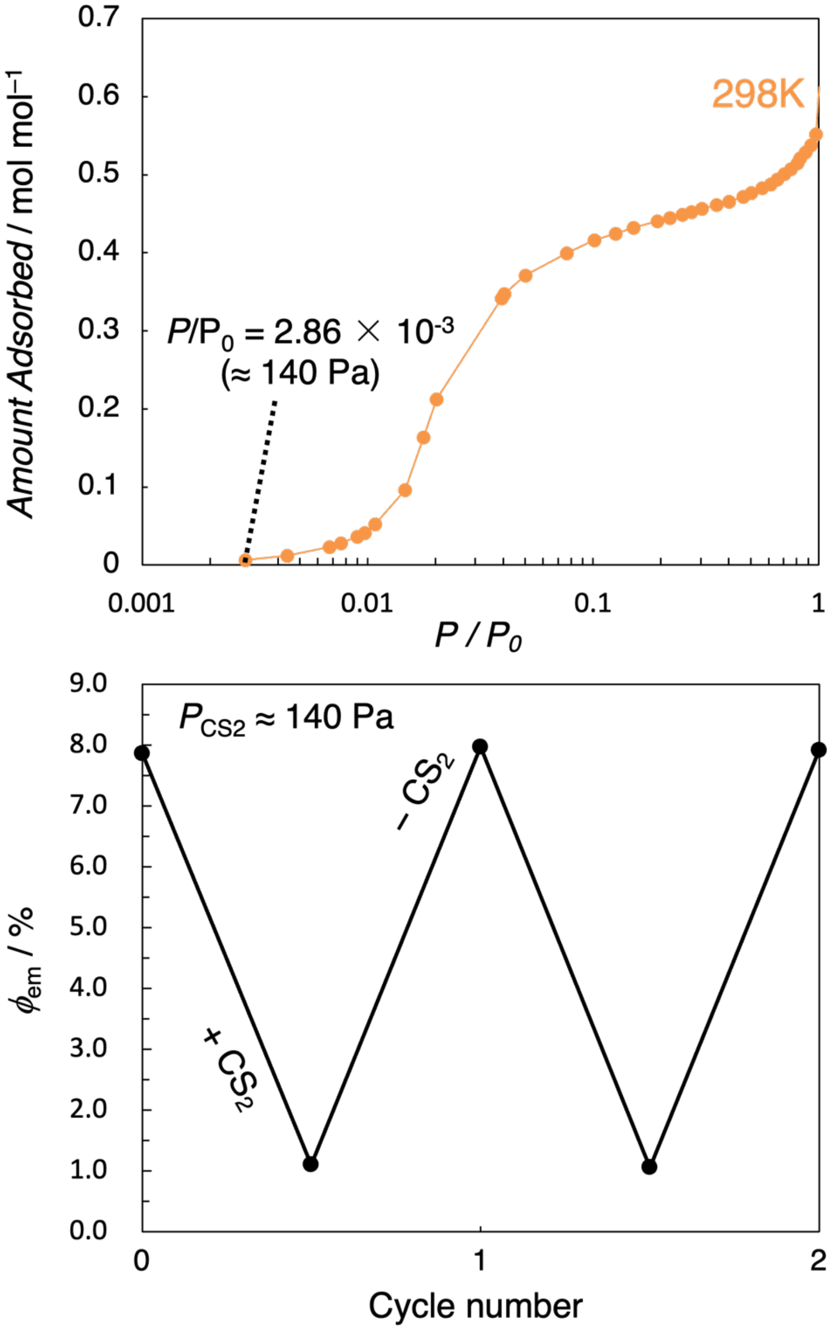

Fig. S12. CS_2 adsorption isotherm of **1** at 298 K and reversible Φ_{em} values from *ex situ* PL measurements under CS_2 at low concentration (P_{CS2} ≈ 140 Pa) at 298 K (λ_{ex} = 365 nm).

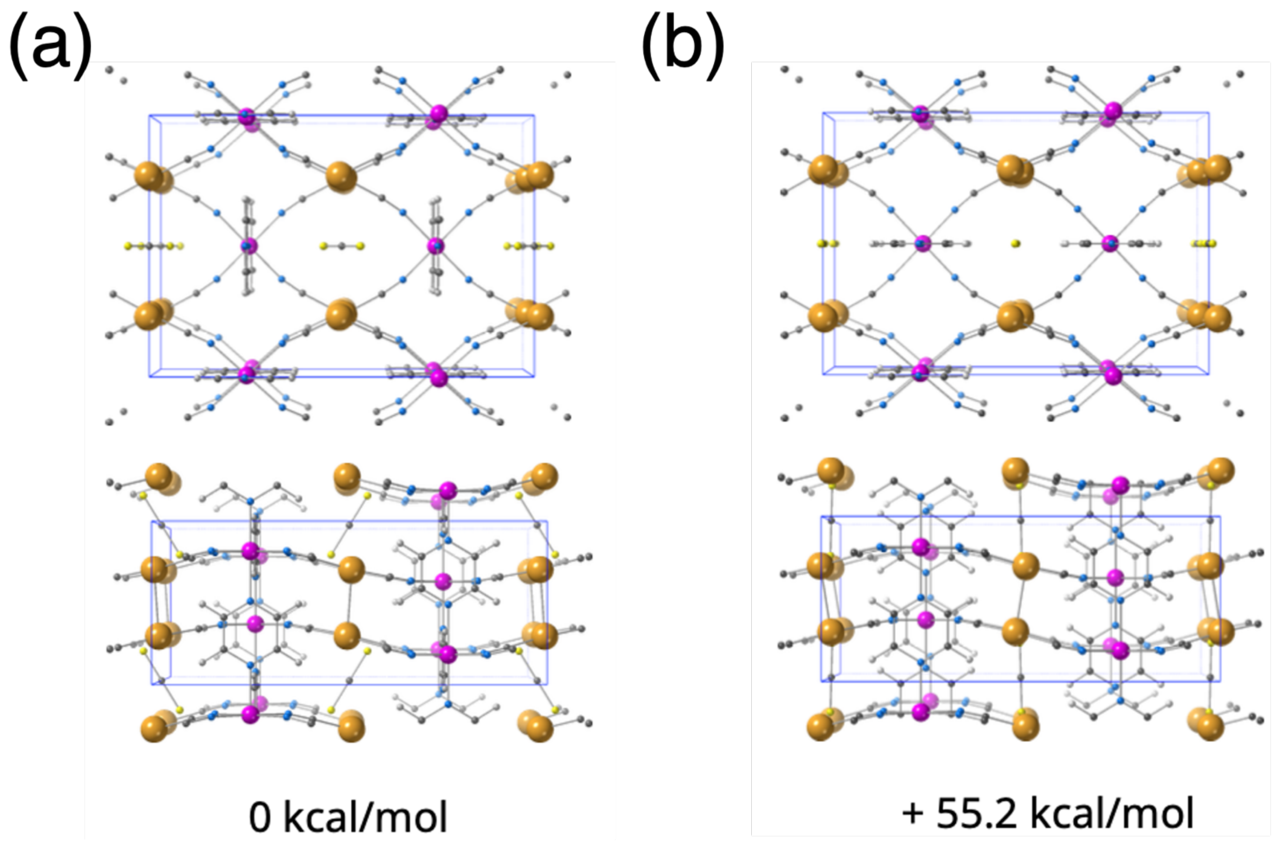

Fig. S13. Comparison of thermal stability of **1_CS$_2$**. (a) optimized structure referring to the SCXRD data at 298 K. (b) optimized virtual structure after pz rotation.

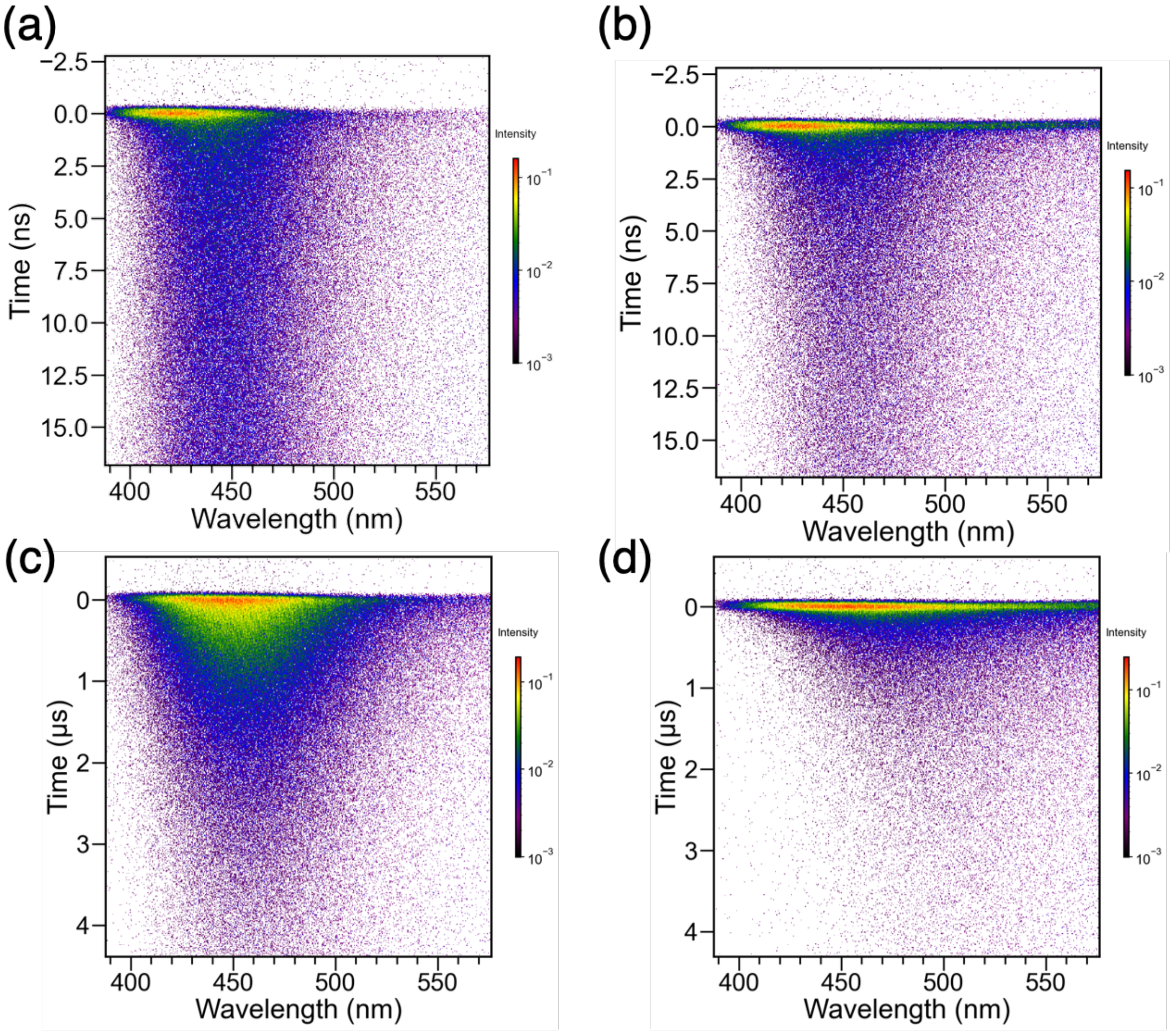

Fig. S14. 2D-plots of *ex situ* TR-PL spectra for (a, c) **1** and (b, d) **1_CS₂** at RT in the (a, b) 20 ns and (c, d) 5 μs time range.

References

[1] H. Pan, J. A. Ritter, P. B. Balbuena, *Langmuir* **1998**, *14*, 6323.
[2] L. Li, R. Krishna, Y. Wang, J. Yang, X. Wang, J. Li, *J. Mater. Chem. A*, **2016**, *4*, 751.
[3] R. D. Spencer, G. Weber, *J. Chem. Phys*. **1970**, *52*, 1654.
[4] CrysAlisPro ver. 1.171.41.89a, Rigaku Oxford Diffraction, 2020.
[5] O. V. Dolomanov, L. J. Bourhis, R. J. Gildea, J. a. K. Howard, H. Puschmann, *J. Appl. Crystallogr.* **2009**, *42*, 339.
[6] G. Sheldrick, *Acta Crystallogr*. **2015**, *71*, 3.
[7] R. Dovesi, A. Erba, R. Orlando, C. M. Zicovich-Wilson, B. Civalleri, L. Maschio, M. Rerat, S. Casassa, J. Baima, S. Salustro, B. Kirtman, *WIREs Comput. Mol. Sci*. **2018**, *8*, e1360.
[8] R. Dovesi, V. R. Saunders, C. Roetti, R. Orlando, C. M. Zicovich-Wilson, F. Pascale, B. Civalleri, K. Doll, N. M. Harrison, I. J. Bush, P. D'Arco, M. Llunell, M. Causà, Y. Noël, L. Maschio, A. Erba, M. Rerat and S. Casassa, CRYSTAL17 User's Manual (University of Torino, Torino, 2017).
[9] J. Laun, D. V. Oliveira, T. Bredow, *J. Compt. Chem*. **2018**, *39*, 1285.
[10] D. V. Oliveira, J. Laun, M. F. Peintinger, T. Bredow, *J. Compt. Chem*. **2019**, *40*, 2364.
[11] J. P. Perdew, K. Burke, M. Ernzerhof, *Phys. Rev. Lett*., **1996**, *77*, 3865.
[12] S. Grimme, *J. Comput. Chem*. **2006**, *27*, 1787.
[13] Y. Zhao, D. G. Truhlar, *Theor. Chem. Acc*. **2008**, *120*, 215.
[14] Gaussian 16, Revision C.01, M. J. Frisch, G. W. Trucks, H. B. Schlegel, G. E. Scuseria, M. A. Robb, J. R. Cheeseman, G. Scalmani, V. Barone, G. A. Petersson, H. Nakatsuji, X. Li, M. Caricato, A. V. Marenich, J. Bloino, B. G. Janesko, R. Gomperts, B. Mennucci, H. P. Hratchian, J. V. Ortiz, A. F. Izmaylov, J. L. Sonnenberg, D. Williams-Young, F. Ding, F. Lipparini, F. Egidi, J. Goings, B. Peng, A. Petrone, T. Henderson, D. Ranasinghe, V. G. Zakrzewski, J. Gao, N. Rega, G. Zheng, W. Liang, M. Hada, M. Ehara, K. Toyota, R. Fukuda, J. Hasegawa, M. Ishida, T. Nakajima, Y. Honda, O. Kitao, H. Nakai, T. Vreven, K. Throssell, J. A. Montgomery, Jr., J. E. Peralta, F. Ogliaro, M. J. Bearpark, J. J. Heyd, E. N. Brothers, K. N. Kudin, V. N. Staroverov, T. A. Keith, R. Kobayashi, J. Normand, K. Raghavachari, A. P. Rendell, J. C. Burant, S. S. Iyengar, J. Tomasi, M. Cossi, J. M. Millam, M. Klene, C. Adamo, R. Cammi, J. W. Ochterski, R. L. Martin, K. Morokuma, O. Farkas, J. B. Foresman, and D. J. Fox, Gaussian, Inc., Wallingford CT, 2016.
[15] J. R. Li, R. J. Kuppler, H. C. Zhou, *Chem. Soc. Rev*. **2009**, *38*, 1477.

Author Contributions

H. Y. designed the project and performed all lab experiments. M. S., T. E., K. M., and K. Onda carried out *ex situ* TR-PL measurements. J.P. and Y. H. conducted computational analysis. S. T. supported breakthrough tests. W. K. helped VT-emission and excitation spectroscopy measurements. K. Otake, and S. K. assisted in *ex situ* SCXRD analyses. H. Y. and H. M. edited the manuscript.
